# *LZTR1:* c.1260+1del Variant as a Significant Predictor of Early-Age Breast Cancer Development: Case Report Combined with *In Silico* Analysis

**DOI:** 10.3390/ijms26146704

**Published:** 2025-07-12

**Authors:** Irena Wieleba, Paulina Smoleń, Ewa Czukiewska, Dominika Szcześniak, Agata A. Filip

**Affiliations:** 1 Department of Clinical Genetics with Cytogenetic Laboratory, Medical University of Lublin, Radziwillowska St. 11, 20-400 Lublin, Poland; ewa.czukiewska@umlub.pl; 2 Laboratory of Molecular Genetics, Medical University of Lublin, Chodzki 1 St., 20-093 Lublin, Poland; p.smolen@nil.gov.pl; 3 Center of Oncology of the Lublin Region St. Jana z Dukli, Jaczewskiego 7 St., 20-090 Lublin, Poland; 4 Medgen Medical Center, Wiktorii Wiedenskiej St 9a, 02-954 Warsaw, Poland; dominika.szczesniak@medgen.pl

**Keywords:** breast cancer, *LZTR1*, *in silico* analysis, phenotype prediction

## Abstract

According to the guidelines of the American Society of Clinical Oncology (ASCO) and the European Society of Medical Oncology (ESMO), the most significant genetic factor in the diagnosis and treatment of breast cancer is the mutation status of the *BRCA1* and *BRCA2* genes. Additional genes with a significant influence on cancer risk were selected for genetic panel screening. For these genes, the disease risk score was predicted to be greater than 20%. In clinical practice, it is observed that rare genetic variants have a significant impact in young patients, characterized by increased pathogenesis and a poorer overall prognosis. The ability to predict the potential effects of these rare variants may reveal important information regarding possible phenotypes and may also provide new insights leading to more efficacious treatments and overall improved clinical management. This paper presents the case of a 38-year-old woman with bilateral breast cancer who is likely a carrier of a pathogenic point mutation in the *LZTR1* gene (*LZTR1*: c.1260+1del variant). With this clinical case report herein described, we intend to display the usefulness of performing detailed molecular tests in the field of genetic diagnostics for patients with breast cancer. Understanding the pathogenesis of hereditary cancer development, which is more predictable and reliable than that of sporadic tumors, will allow for the discovery of hitherto hidden intrinsic signaling pathways, facilitating replicable experimentation and thereby expediting the discovery of novel therapeutic treatments.

## 1. Introduction

### 1.1. Genetics of Breast Cancer

Breast cancer has been identified as the most prevalent type of cancer in terms of morbidity and mortality rates among women on a global scale. Educational programs and well organized diagnostics including screening programs, together with additional treatment regimens, significantly improve the quality of medical care on female patients with breast cancer. However, according to predictions, the frequency of breast cancer is increasing at an annual rate. It has been hypothesized that this may be related to high environmental impact on the disease’s ethicology [1,2]. According to the results presented by Murthy et al., early diagnosis is less effective in reduction in mortality rates of malignancies than early prevention [3]. Nevertheless, further investigation into high-impact prognostic factors within appropriate evaluation may improve clinical management in oncology.

**Figure 1:**
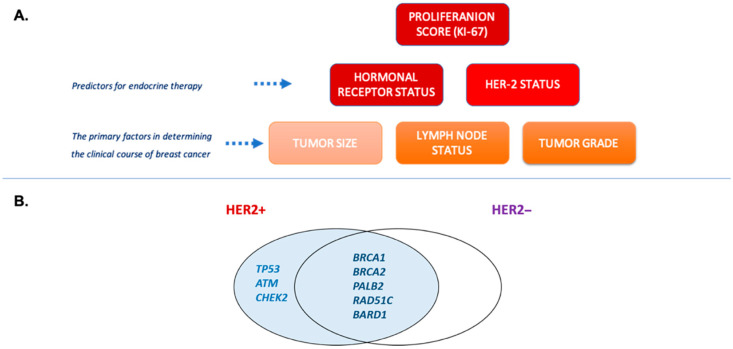
(**A**) Breast cancer risk factors used in therapeutic decisions. Genetic molecular testing is used for investigation of hereditary risk factors and calculation of negative prognostic score. (**B**) Germline mutations as potential predictors of HER2 expression. Based on meta-analysis data by Rowlands et al. [12].

Individuals at risk of hereditary breast and/or ovarian cancer should undergo regular ultrasonographic diagnostics on an annual basis. In addition, patients deemed to be at high risk may require further evaluation through mammography and/or magnetic resonance imaging of the breast. In cases where a definitive diagnosis is uncertain, the performance of a diagnostic biopsy may be considered. The treatment strategy is predicted by disease stage, according to World Health Organization (WHO) guidance and the hormonal status of malignant cells. The gold standard in the treatment of breast cancer is surgery involving partial resection or mastectomy. In cases where patients are confirmed to be at high risk of developing breast cancer, as determined by the molecular testing of high-risk genes, prophylactic mastectomy may be a recommended course of action. Systematic treatment includes chemotherapeutic agents and/or endocrine therapy. This treatment option could be provided in the preoperative or postoperative management. According to the current guidance from the National Comprehensive Cancer Network (NCCN), patients diagnosed with locally advanced or inoperable breast cancer (cN2 axillary nodes; cN3 regional lymph node disease; cT4 tumors) may be eligible for preoperative systemic treatment [4]. A significant role in the prediction of disease course is played by the disease advancement stage and expression level of the following three main hormonal receptors: human epidermal growth factor receptor (HER-2), estrogen receptor (ER), and progesterone receptor (PR) (breast cancer risk factors are described in Figure 1) [5].

Approximately 10–15% of breast cancer cases are corelated with hereditary predispositions, while the majority of diagnosed cases remain sporadic [6]. In instances of hereditary breast cancer, the occurrence of the condition within a family unit is indicative of a genetic predisposition, often attributable to specific mutations in relevant genes. The highest risk of breast cancer is determined for carriers of *BRCA1* and *BRCA2* mutations. Germline mutations in these genes have been demonstrated to increase the risk of breast and ovarian cancer syndrome development. A comprehensive analysis of the germline mutations present in a large sample population has led to the identification of additional variants associated with the disease stage. Genes that are characterized by high penetrance and a predisposing effect to breast cancer include the tumor protein 53 gene (*TP53*), phosphatase and tensin homology gene (*PTEN*), cadherin1 gene (*CDH1*), and serine/threonine kinase 11 gene (*STK11*). In the category of moderate-penetrance genes, the following have been identified: ataxia-telangiectasia mutated gene (*ATM*), BRCA1-associated RING domain 1 (*BARD1*), checkpoint kinase 2 (*CHECK2*), partner and localizer of BRCA2 gene (*PALB*2), *RAD51C* and *RAD51D* genes [7,8]. These genes are frequently incorporated into gene panel tests, which are used in diagnostic screening. An essential part of genetic testing is the knowledge about the genetic homogeneity of the population. The frequency of specific alleles could be different in countries with a more or less homogeneous population. Poland as a country is characterized by a high degree of homogeneity. Consequently, the interpretation of genetic test results should be individualized. It highlights the necessity of preparing specific prediction and diagnostic categories that will consider genetic homogeneity [9,10]. The LZTR1 gene has not yet been incorporated into the standard gene panel for hereditary breast–ovarian cancer syndromes, because of the low frequency and unconfirmed status in disease development [11].

The *LZTR1* gene is included into expanded NGS oncology panels because of confirmed hereditary cancer syndrome risk. Additionally, rare variants of *LZTR1* were confirmed for different cancer types (glioblastoma, hepatocellular, esophagogastric, colorectal, and lung cancers), including breast cancer. Functional studies on cell cultures and animal models revealed possible oncogenic mechanism driven by *LZTR1* mutation. In the Gen2Phenotype database, information about *LZTR1* involvement in hereditary breast carcinoma is described (ID: G2P03479) [13]. But there is no clear information about gene variants related to the disease.

*LZTR1* pathogenic variants are mainly related to Schwannomatosis and Noonan syndrome. The median age for symptoms initiation is 30 years old, while disease diagnosis is normally confirmed above 40 years old [14]. Based on these data we can hypothesize that patients with rare *LZTR1* pathogenic variants could manifest disease symptoms under 50 years old. For proper phenotype prediction, epidemiological studies are highly needed. TCGA and related datasets could be used to support the investigation of *LZTR1* mutation frequency in breast cancer patients. The open access dataset from Breast Invasive Carcinoma (TCGA, Firehose Legacy) at cBioPortal presents *LZTR1* as a more typically mutated gene in breast invasive ductal carcinoma and/or breast invasive lobular carcinoma. Mutation frequency was less than 2%. In comparison, confirmed variant prevalence for germline genetic variants of the *TP53* gene in patients diagnosed with breast cancer under 40 years old is about 5% [15]. People of white race more likely carried the mutation in the *LZTR1* gene and were frequently diagnosed under 40 years old [16].

According to gnomAD data (accessed on 27 June 2025), *LZTR1* c.1260+1del variant total allele frequency is 0.000001888, which is in the range 10^−4^ > (0.1888 × 10^−5^) > 10^−6^. Based on the Bonferroni testing threshold for independent genetic variants, an appropriate *p* value is <5 × 10^−7^. Hence, the currently available data about the described variant should be improved. Population allele frequency from Human Genome Diversity (HGDP) is not available for this variant.

### 1.2. Biological Role of LZTR1 Protein

**Figure 2:**
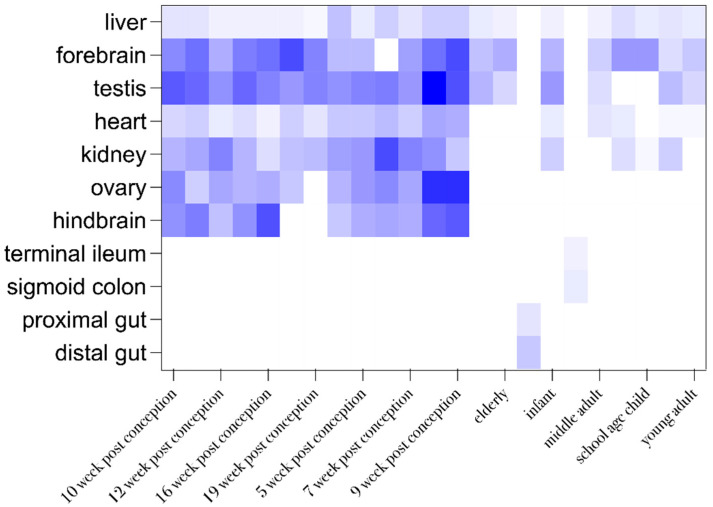
Organ and developmental stage-dependent expression of LZTR1. Data were excluded from the Expression Atlas in tabular view. Row statistics and heat-map generation were performed by means of Graphpad prism 10. A higher color gradient indicates higher frequency of reported protein expression.

*LZTR1* is a tumor suppressor gene that encodes the leucine zipper-like post-translational regulator (LZTR1) protein which belongs to BTB-Kelch superfamily [17]. LZTR1 is a Golgi protein reported to be involved in apoptosis [18]. According to the data available in UniProt, the LZTR1 protein (Q8N653) indirectly regulates the ubiquitination of Ras family proteins. Hence, this protein plays a role of negative regulator of Ras/KRAS, N-RAS and H-RAS proteins, because of the extenuation of Ras/MAPK signaling [19]. Functionally active LZTR1 protein forms a complex with cullin-3 ubiquitin ligase complex (CUL3), which then promotes the ubiquitination of Ras proteins [20]. Six beta-sheet blades or repeats form Kelch’s domain, which is located on the N-terminus and two BTB-domains separated by BACK domains on C-terminus of LZTR1 [21]. BTB domains have a conservative structure, and they could interact in homodimerization. Besides involvement in the incorporation into Golgi apparatus, BTB domains possibly play a significant role during LZTR1-E3-CUL3 complex formation and its stabilization [20]. Kelch domains are responsible for substrate binding and regulating E3 activity [22]. These domains have the ability to bind different cellular proteins. This indicates the possible involvement of Kelch proteins in the development of different diseases [23,24]. During prenatal development, the LZTR1 protein is expressed in the lung, liver, kidney, brain, and heart. The tissue-specific expression of LZTR1 protein depends on the developmental stage (Figure 2). In adults, this protein is present mainly in vessels and the immune system [17,19]. According to the transcriptomic Expression Atlas, there is no significant LZTR1 protein expression in the breast. However, *LZTR1* expression on a transcriptomic level was confirmed in patients (both sexes) with breast cancer, but its status was described as not significant in disease pathogenesis because of its insignificant frequency in the study population. Additional information in the Expression Atlas for studies on different cell lines and cell types revealed the medium range expression of LZTR1 on transcriptomic and proteomic levels for selected malignant and normal cell lines [25].

The manuscript presents the case of a Polish 38-year-old woman with bilateral breast cancer and a family history of cancer. The patient was found to be a carrier of a point mutation in the leucine zipper-like post-translational regulator 1 gene (*LZTR1*). At present there is no information available concerning the molecular consequences of this genetic variant. In our research we used bioinformatic tools to predict variant pathogenicity to shed more light to the molecular basis of cancer development in this case. The knowledge about genetic background could result in the discovery of a new prediction factor classification and highlight the necessity of gene variant studies in cancer.

In this paper we present a case report of a breast cancer patient combined with the bioinformatical study of possible phenotypic consequences of (*LZTR1* c.1260+1del) germline mutation.

### 1.3. Clinical Case Description: Clinical Examination

**Figure 3:**
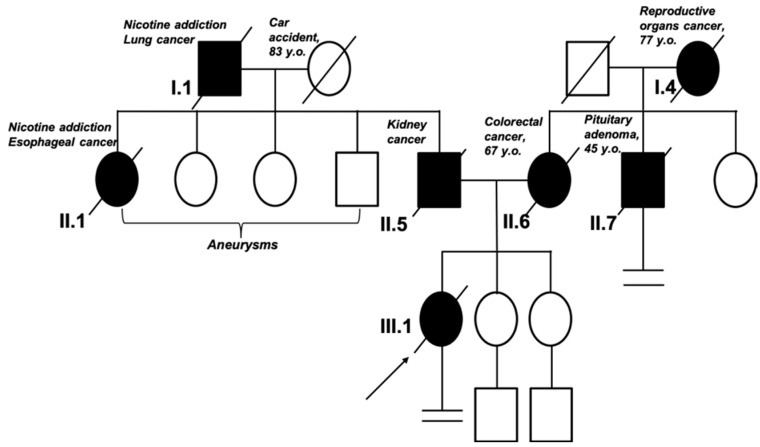
Pedigree of the patient’s family. The arrow indicates the proband. According to the family history, in the first generation, the grandfather of the proband in the paternal line died of lung cancer. In the second generation, the father of the proband and his sister (the proband’s aunt) were diagnosed with cancer; the siblings were also diagnosed with aneurysm. In the first generation in the maternal line, there was no evidence of cancer. In the second generation, the proband’s mother and uncle were diagnosed with cancer at a young age (<50 y/o).

A 38-year-old female patient was reported to the genetic outpatient clinic at the MEDGEN with a diagnosis of bilateral breast cancer (synchronous cancer). She was not treated for chronic diseases before. Medical interview revealed the following: no childbirth; negative status of oral hormonal contraception use and probability of hereditary cancer family history. The patient’s family history was burdened with cancer diseases as follows: mother diagnosed with colon cancer at the age of 62; father suspected of having kidney cancer at the age of 47, deceased (Figure 3). An oligobiopsy of the left breast tumor and pathologically changed lymph nodes was performed. The histopathologist conclusion was invasive breast cancer, NOS (No Otherwise Specified according to the WHO 2018 classification); the pathologic grade of malignancy according to the Bloom-Richardson Scale in the Elston-Ellis modification was (3 + 2 + 1 = 6) G2. The immunohistochemical examination of the tumor sample showed a positive result for the expression of Estrogen Receptor (ER), 100% (Allred’s scale: PS5+IS3=TS8), and Progesterone Receptor, (PGR) 100% (Allred’s scale: PS5+IS3=TS8), but negative results for HER2 receptor, 0%. The proliferation index was high positive, Ki67 > 30%. The histopathological subtype was qualified as Luminal type B, HER2 negative, *infiltratiocarcinomatosa,* stage cT4N2Mx according to the TMN scale. The histopathological test result of the oligobiopsy of the right breast was invasive cancer without special type (NST G2), type Luminal A, ER, PR positive 100%, HER2 negative; Ki67 < 14%. The clinical stage was cT1bN0Mx. Approximately 14 days after the biopsy, the patient underwent CT scan of the chest, abdominal cavity, and pelvis, and the examination revealed metastases in the left axillary nodes; several slightly blurred and poorly saturated sclerotic foci were scattered in the skeleton—mainly in the spine and pelvic girdle—the largest focus of 10 mm in diameter in the L3 vertebral body was observed, which most likely were metastatic lesions. In-depth diagnostics in the form of bone scintigraphy revealed numerous disseminated bone metastases (located in the spine, ribs, pelvis, left humerus, and femur). The patient was not qualified for surgery. She received the following treatment: zoledronic acid 4 mg intravenously, an inhibitor of cyclin-dependent kinases type 4 and 6 (CDK4 and CDK6) Abemaciclib 2 × 150 mg orally, hormonal treatment Etruzil 2.5 mg daily, and Goserelin 3.6 mg subcutaneously every 28 days.

Unfortunately, despite treatment, rapid progression of the disease was observed. The patient died 6 months after the therapy was initiated.

## 2. Results

### 2.1. Molecular Diagnostic

The c.1260+1del variant was identified in one allele of the *LZTR1* gene (heterozygous genotype). According to gnomAD this variant is a frameshift mutation without germline classification (22-20992903-AG-A, GRCh38). This variant is not described in the ClinVar database. *LZTR1* variant (*LZTR1*: c.1260+1del) examination by the Franklin suggested variant classification as a likely pathogenic [26]. There is no report concerning the clinical significance of this *LZTR1* variant in the Orphanet database.

### 2.2. Bioinformatical Analysis of the Gene Variant

#### 2.2.1. Analysis of the Gene and Transcript Structures Combined with *LZTR1:* c.1260+1del Pathogenicity Assessment

**Figure 4:**
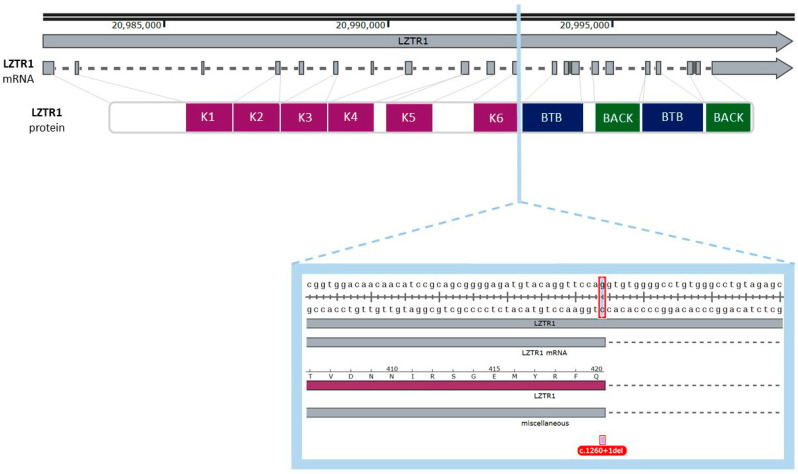
Graphical representation of the described variant (c.1260+1del) of the *LZTR1* gene. One nucleotide deletion is localized in the eleventh exon of the gene, and it spans the donor splice site. The deleted nucleotide is a part of a codon for glutamine (Q). The figure was created using SnapGene Viewer (v8.1.0).

The *LZTR1* gene is located on chromosome 22q11.21. The open reading frame (ORF) includes 21 exons. The gene includes misc_features sequences which could play a significant regulatory role. One of the misc_features sequences is defined as lymphoblastoid silent region and was described as a silencer of GM12878 lymphoblastic cells [27,28]. The biological impact of others is not described. The total number of misc_feature regions in the *LZTR1* gene is seven. One of the described regions overlaps with the mutation site in exon no 11. According to gnomAD, the c.1260+1del variant was described in three Europeans (non-Finnish), two males and one female. This variant was described in terms of genomic and transcriptomic levels. The deletion of the single nucleotide results in frameshift mutation with high probability of loss of function (LoF), but it was not specified as a splice site mutation according to gnomAD data (accessed on 6 March 2025). There is no phenotype description for *LZTR1*: c.1260+1del variant. The graphical presentation of the analyzed variant is presented in Figure 4.

**Figure 5:**
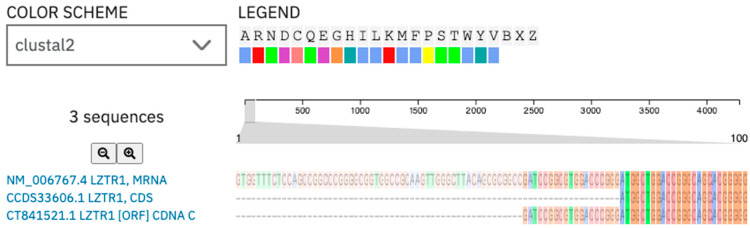
Alignment of the *LZTR1* mRNA sequence [NM_006767.4] and its CCDS sequence [CCD33606.1]: the ccds sequence is a consensus coding sequence. The c.1260+1del variant description in GnomAD is referenced to the ccds sequence of the human LZTR1 gene. Herein, those ccds sequences were used for further analysis.

In the subsequent stage we examined possible transcript and protein structures. It is noteworthy that the mutation is related to the deletion of the last nucleotide in the exon, which may result in splicing impairment. The 1260 position in mRNA was different from the position described in gnomAD. To find the reason for the position variance, we compared both sequences in the Clustul Omega alignment tool. (v1.2.4.) The result showed sequence similarity in whole length of the ccds sequence for the *LZTR1* gene and this is presented in Figure 5. The consensus coding sequence was predicted with a higher prediction grade of protein-coding sequences and is currently integrated into the main datasets [29,30].

**Figure 6:**
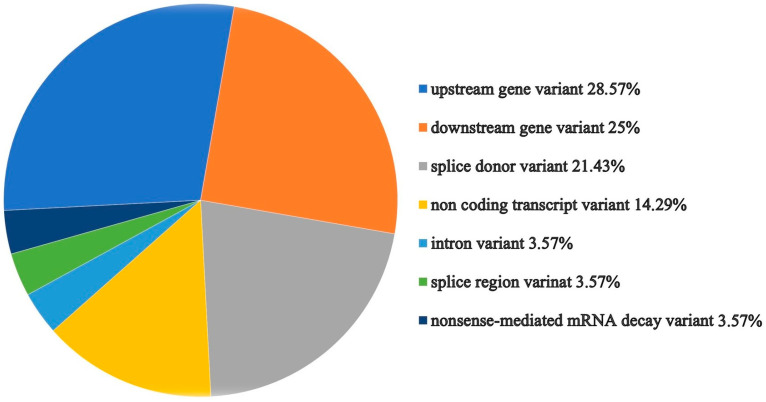
Graphical presentation of the LZTR1 variant effect prediction by the Ensemble tool [31].

As a result of the analysis by the Variant Effect Predictor tool from Expasy, the majority of transcripts were classified as upstream gene variant, downstream gene variant, or splice donor variant (Figure 6).

#### 2.2.2. Analysis of Genetic Variant Influence on Splicing

**Figure 7:**
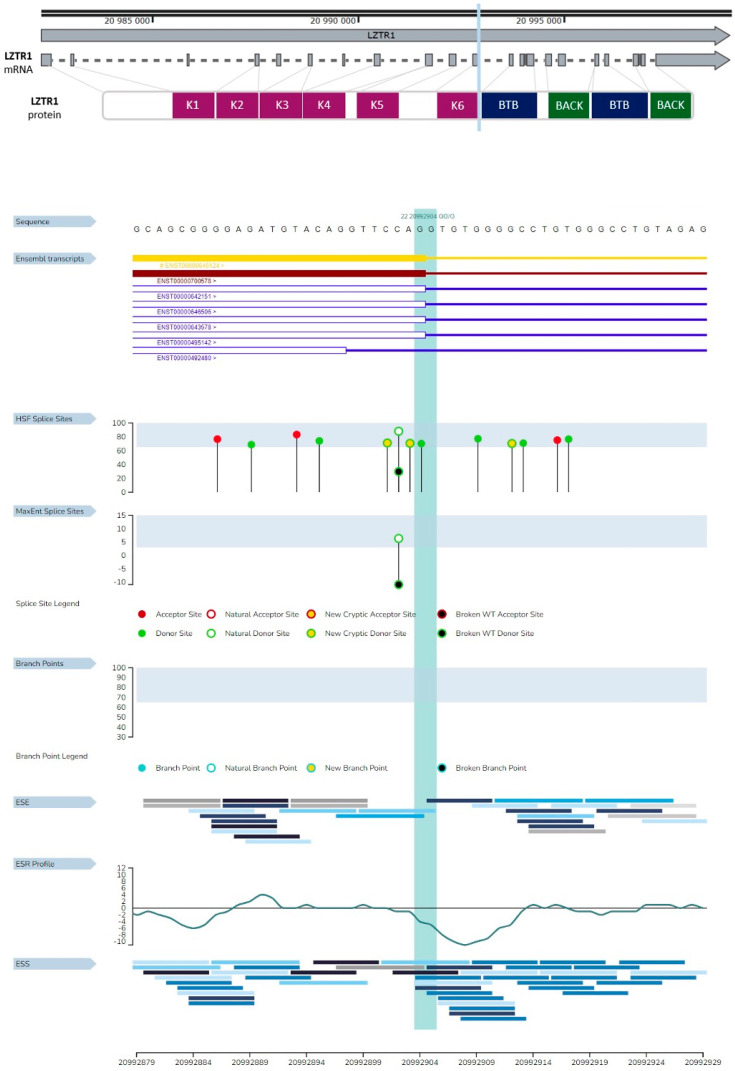
Consequences of *LZTR1* c.1260+1del on splicing. The region marked with light blue represents the spot of the deletion. HSF splice site area presenting three new cryptic donor sites.

HSF GenOmnis analysis of *LZTR1* c.1260+1del revealed three new cryptic donor sites. The deletion does not span the natural donor site of splicing (Figure 7). These results suggest few possibilities of gene transcript formation, outlined as follows: the possibility of frameshift or selection of one of the new cryptic donor sites during splicing. Analysis by MutationTaster revealed splice site changes and frameshift. The prediction of frameshift is similar to the HSF GenOmnis results (Table 1). Variant examination by SpliceAI confirmed the loss of a donor splicing site and the gain of a new donor splicing site. Results from the SpliceAI analysis are available at the link (https://spliceailookup.broadinstitute.org/#variant=chr22-20992903-AG-A&hg=38&bc=basic&distance=500&mask=0&ra=0) (accessed on 12 March 2025) [32].

Examination of the splice sites by the BDGP: Splice Site Prediction by Neural Network showed the same splice sites for the analyzed variant and reference sequence of *LZTR1* gene (Supplementary Materials File S2). This result confirms the frameshift mutation type as a molecular effect of the analyzed variant.

#### 2.2.3. Gene Variant Transcript Prediction, Protein Structure Design, and Evaluation

**Figure 8:**
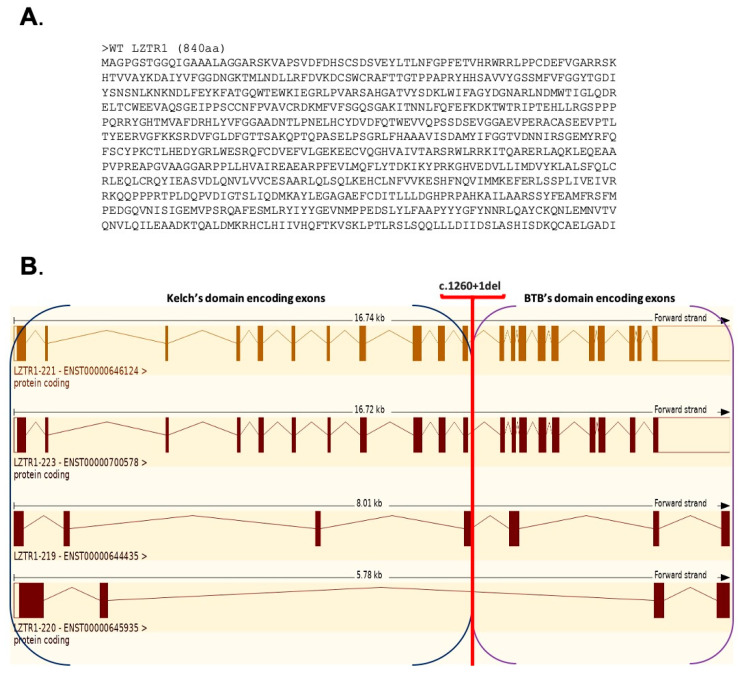
(**A**) Amino acid sequence for the WT LZTR1 protein (840 aa, transcript variant LZTR1-221). (**B**) Graphical comparison of 4/21 protein coding transcript variants for LZTR1 gene.

Data available in the Ensemble database define four protein coding transcript variants for the WT LZTR1 protein; their structures are presented in Figure 8.

Prosite and Smart tools distinguish only the BTB domains in the analyzed amino acid sequences of WT, MT1, MT2, MT3, and MT4 proteins. The Prosite tool has been used for the identification in analyzed sequences of the Kelch and BTB domains. The results of the analysis performed by Prosite have the highest similarity score with the information about the WT LZTR1 protein included in databases. For this reason, data collected from Prosite were used to compare structure elements of the predicted proteins (Table 2).

**Figure 9:**
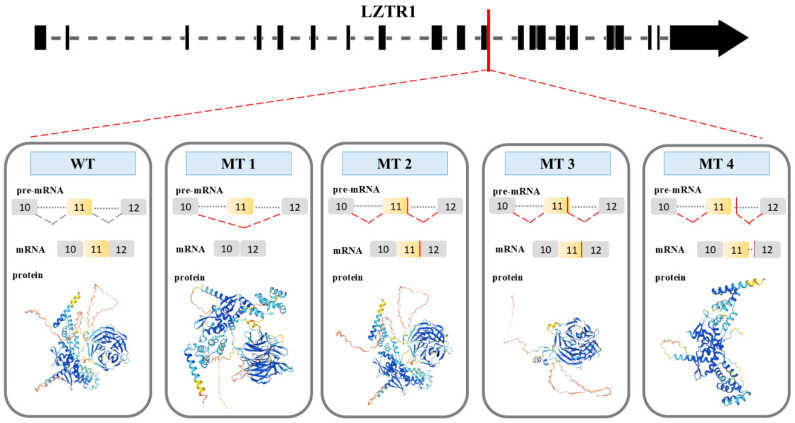
Four possible consequences (MT1, MT2, MT3, and MT4) of splice site mutation of LZTR1 gene (*LZTR1* c.1260+1del). WT represents splicing in the wild-type gene. MT 1 shows exon skipping as a consequence of the mutation. Donor splice site in the MT 2 sequence: ACAGGTTCCATT. Spot of the splicing is in 11th exon sequence. Donor splice site in the MT 3 sequence: GGTTCCATTTCT. Spot of the splicing is located within 11th exon of the gene. For MT4, the donor splice sequence is TGTGGGGCCTGT, and the spot of the splicing is located within the intron between exon 11th and 12th.

WT protein was constructed by using the splice site in each exon of *LZTR1*. InterPro analysis showed four Kelch motifs, six Kelch type beta propellers, four Kelch smart, two BTB domains, and two BTB back domains. In the available databases, the WT LZTR1 structure consist of six Kelch motifs (Figure 9) [33]. The Prosite tool was used for the preliminary comparison of protein structures.

During the splicing of the MT1 pre-mRNA, the sequence of exon 11th is deleted. As a result, MT1 protein has two BTB domains and one Kelch domain which consists of two Kelch motifs and four Kelch type beta propellers. For the splicing of the MT2, a new donor splice site on the 11th exon was used (ACAGGTT**C**CATT). MT2 protein has a BTB domain and Kelch domain with three Kelch motifs and six Kelch type beta propellers (Figure 9). MT1 and MT2 had a high possibility of preserved N- and C-terminus structure, which is like the WT protein structure. The protein structures of MT1 and MT2 do not have the same number of Kelch motifs like the WT protein. Consequently, the lack of a Kelch motif amino acid sequence is the result of a dysfunction of the Kelch domain. The Kelch domain is not responsible for the initial steps during the binding substrate ubiquitination process. The results presented by C. L. Philips et al. show that every motif in the Kelch domain is necessary for proper protein function. The lack of one of the motifs could result in protein malfunction [34].

**Table 2:** Determination of protein family and domains by the InterPro tool.

	WT	MT1	MT2	MT3	MT4
Family	Leucine-zipper-like transcriptional regulator	Leucine-zipper-like transcriptional regulator	Leucine-zipper-like transcriptional regulator	Leucine-zipper-like transcriptional regulator	Leucine-zipper-like transcriptional regulator
Domains	4 Kelch motifs 6 Kelch type beta propellers 4 kelc_smart BTB1_POZ_LZTR1 BACK1_LZTR1 BTB2_POZ_LZTR1 BACK2_LZTR1	2 Kelch motifs 4 Kelch type beta propellers 4 kelc_smart BTB1_POZ_LZTR1 BACK1_LZTR1 BTB2_POZ_LZTR1 BACK2_LZTR1	3 Kelch motifs 6 Kelch type beta propellers 4 kelc_smart BTB1_POZ_LZTR1 BACK1_LZTR1 BTB2_POZ_LZTR1 BACK2_LZTR1	3 Kelch motifs 6 Kelch type beta propellers 4 kelc_smart	BTB1_POZ_LZTR1 BACK1_LZTR1 BTB2_POZ_LZTR1 BACK2_LZTR1
Link for the analysis results	[35]	[36]	[37]	[38]	[39]

In MT3 the new donor splice site within exon 11th (GGTTCC**A**TTTCT) was used for splicing. The MT3 protein structure includes N-terminus and C-terminus domain. The MT3 protein consists of the Kelch domain with three Kelch motifs and six Kelch type beta propellers (Figure 9). The absence of BTB domains prevents MT3 from interacting with the E3 ligase complex. Consequently, proteins bound to the Kelch domain will not be targeted for degradation by the proteasome. Furthermore, the absence of one of the Kelch motifs results in domain dysfunction.

The new donor splice site (TGTGGGGCCTG), which is located in the intron between exons 11 and 12, results in the production of the MT4 protein. This protein contains two BTB domains (Figure 9). The MT4 protein has defined N- and C-terminal domains. Although the MT4 protein consists solely of the BTB domain and can interact with the E3 ligase complex. It cannot tag any protein for degradation due to the absence of a Kelch domain. The partially preserved structure of MT1, MT2, MT3, and MT4 suggests a LoF mutation resulting in the absence of a functional protein.

### 2.3. Analysis of Gene-Related Phenotypes

The results of the analysis of already reported data are presented in Table 3. Phenotypes related to *LZTR1* variants mainly include Noonan Syndrome and Schwannomatosis. Only the Orphanet database has additionally reported the glioblastoma phenotype.

### 2.4. Analysis of Protein–Protein Interactions

Databases of protein–protein interaction were used to identify potential functional partners of LZTR1. The STRING database (accessed on 22 May 2025) identified several proteins with a high confidence score of interaction with the LZTR1 protein, outlined as follows: CUL3 (Cullin-3), KLHL22 (Kelch-like protein 22), KRAS (GTPase Kras), KLHL3 (Kelch-like protein 3), ZSWIM8 (Zinc finger SWIM-type containing 8), GAN (Gigaxonin), KLHL21 (Kelch-like protein 21), KLHL8 (Kelch-like protein 8), KBTBD8 (Kelch repeat and BTB domain-containing protein 8), and KLHL7 (Kelch-like protein 7).

Analysis performed with the Genemania tool predicted several interactions with LZTR1, outlined as follows: RIT2 (Ras-like without CAAX2), SLC44A1 (Solute carrier family 44 member 1), BMPR1B (bone morphogenetic protein receptor type 1B), KLHDC3 (Kelch domain containing 3), KLHDC4 (Kelch domain containing 4), RIT1 (Ras-like without CAAX1), SCYL2 (SCY1-like psudokinase2), LRCH4 (leucin-rich repeats and calponin homology domain containing 4), HSPG2 (heparan sulfate proteoglycan 2), PBXIP1 (PBX homeobox interacting protein 1), SGSM3 (small G protein signaling modulator 3), PPIL2 (peptidylpropyl isomerase-like 2), LTBP3 (latent transforming growth factor beta binding protein 3), AIFM3 (apoptosis inducing factor mitochondria associated 3), ANKRD11 (ankyrin repeat domain 11), RBM10 (RNA binding motif protein 10), KLHL22 (Kelch-like family member 22), ASCC2 (activating signal coinfegrator 1 complex subunit 2), TUBGCP4 (tubulin gamma complex-associated protein 4) and CABIN1 (calcineurin binding protein 1).

### 2.5. Protein Homology Analysis

**Figure 10:**
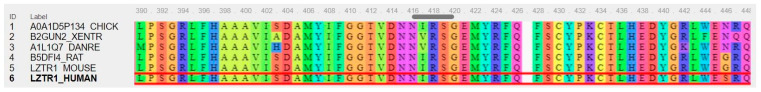
Alignment of multispecies amino acid sequence of leucine zipper-like post-translational regulator 1 (LZTR1). The presented amino acid region represents the spot of the mutation in the analyzed variant. The human LZTR1 protein is marked in the red box. The figure was created using PipeAlign2.

LZTR1human protein (UniProt ID: Q8n653) has homologs with LZTR1 proteins from *Rattus norvegicus* (RAT; UniProt ID: B5DFI4), *Mus musculus* (MOUSE; UniProt ID: Q9CQ33), *Danio rerio* (DANRE; (UniProt ID: A1L1Q7), *Gallus gallus* (CHIC; UniProt ID:A0A1D5P134), and *Xenopus tropicalis* (XENTR; B2GUN2) (Figure 10). The multispecies alignment showed a high score of compatibility. The amino acid sequence which spans the mutation site has a conserved domain.

### 2.6. Model Organism’s Phenotype Analysis

Model organisms with a high *LZTR1* homology score were selected for phenotype examination. The ZFIN database marked two phenotypes related with the *LZTR1* gene [40]. These phenotypes include cranial vasculature malformation and cardiac ventricle hypertrophy. Alliancegenome showed for *Lztr1 Mus musculus* gene phenotypes related with abnormal cranium and facile morphology, abnormal heart echocardiography, cardiac hypertrophy, increased cell proliferation, and premature death [41]. Data about *Xenopus tropicalis* and *Rattus norvegicus* with *Lztr1* and *Lztr1* variants, respectively, showed the following phenotypes: cardiovascular, central nervous, and gastrointestinal system disease; thyroid disease; benign neoplasm; cancer and pre-malignant neoplasm. As a result of *Lztr1* knockout mutants, lethality is observed because of cardiovascular abnormalities [42]. *Lztr1* autosomal dominant mutation induces Noonan syndrome-like phenotypes in mice [43]. Research presented by J. W. Bigenzahn et al. shows a Ras-dependent gain-of-function phenotype in *Drosophila* after *Lztr1* knockdown [44].

## 3. Discussion

Hereditary predispositions occur in 10–15% of breast cancer cases. Most of them are associated with variants of the *BRCA1* and *BRCA2* genes which are involved in the mechanisms of DNA repair. Furthermore, other gene variants are also associated with familial cancers [6]. The *LZTR1* gene has not yet been included in the main gene panel used in hereditary breast–ovarian cancer syndrome testing. Selected gene variants were confirmed in hereditary cancer syndrome with a higher risk of resistant *KRAS* mutated lung adenocarcinoma, colorectal and esophageal cancers, hepatocellular carcinoma, and glioblastoma [11,45]. The knowledge about the genetic background of such variants could result in the discovery of a new prediction factor classification. These kind of studies highlight the necessity of gene variant studies in cancer.

The aim of the study was to describe the case of a patient with a genetic variant of *LZTR1* c.1260+1del and to determine the phenotypic effect of this germline variant. Moreover, by the *in silico* studies we wanted to evaluate the molecular mechanism of cancer development. This part of the study focused on finding a correlation between familial occurrence of cancer and detected genetic variants of *LZTR1.*

The presented prediction of transcripts and protein structures of *LZTR1* c.1260+1del distinguished four possible proteins. The partially preserved structure of all of the predicted proteins suggested a LoF mutation of the analyzed *LZTR1* variant. This kind of mutation results in the lack of a functionally active protein. Loss of function mutations in the *LZTR1* gene were described in one-fifth of glioblastoma cases. This type of malignancy commonly occurs in both children and adults and remains to be related with hereditary cancer syndrome [46]. Phenotypes related to germline mutation of analyzed gene are heterogenous. Commonly observed features include dysmorphology and hypermobility. The clinical phenotypes related to *LZTR1* variants are mostly demonstrated as nerve sheath tumors. The role of germline LoF *LZTR1* mutations in other tumors remains unknown because of the absence of typical clinical phenotypes. It is still unknown whether these *LZTR1* LoF variants could be associated with RASopathies [47].

The analysis of the protein–protein interactions of LZTR1 demonstrated this protein’s cooperation with CUL3 and KRAS. The LZTR1-CUL3 complex is a negative regulator of EGFR-AXL level. Decreased ubiquitination provides the accumulation of tyrosine kinases. Ko et al. reported the susceptibility of *LZTR1*-mutated tumors to EGFR inhibitors (58). The lack of LZTR1-CUL3 complex results in the accumulation of EGFR and AXL and exhibited specific vulnerability to EGFR and AXL co-inhibition. The investigation presented by Ko et al. revealed the possible effectiveness of already existing drugs (Osimertinib and afatinib) in the treatment of *LZTR1*-mutated cancers [48]. Coexisting germline mutations in the *LZTR1* gene could trigger tyrosine kinase inhibitor (TKI) resistance in solid tumors [49].

Tissue-specific and age-related variability in *LZTR1* expression possibly targeted patients’ phenotypes. *LZTR1* knock-out in lung cancer cells described by Abe et al. confirmed its positive impact on the overactivation of RAS/MAPK kinases, which enhance cell proliferation. Abe had also proved LZTR1 involvement in collagen synthesis, hence protein absence influences extracellular matrix remodeling and possibly promotes epithelial–mesenchymal transformation [50]. Biegenzahn et al. discovered that cancer cell sensitivity against selected drugs depends on a genetic variant of the *LZTR1* gene. Additionally, this group revealed that, in contrast to Kelch’s domain mutations, BTB/BACK mutations did not disrupt cellular sensitivity to imatinib [44].

The present study showed a high score of LZTR1-domain conservation among different organisms. For this reason, the analysis of studies with model organisms could clarify LZTR1 variant function in cancer development. Abe et al. demonstrated that *Lztr1* autosomal dominant mutation induces Noonan syndrome-like phenotypes in mice [43]. Research presented by J. W. Bigenzahn et al. shows a Ras-dependent gain-of-function phenotype in *Drosophila* after *Lztr1* knockdown [44]. The study performed by Pae et al. showed that in *Drosophila* the CUL3 plays a crucial role in targeting Torso/RTK degradation [the homolog of the torso in humans is a platelet-derived growth factor receptor beta (PDGFRB)]. It remains the possible source of oncogenic signaling [51]. Moreover, in studies with *Drosophila melanogaster*, Zipper et al. demonstrated that the loss of function variant of *Lztr1* results in increased cell proliferation, RAS pathway activation, and induction of apoptosis [52]. The lack of normal LZTR1 protein leads to alterations in the RAS protein level. Higher amounts of RAS protein promote the overactivation of the RAS/MAPK pathway, which is manifested in abnormal cell proliferation and the differentiation of affected cells [19,47,53,54]

The presented clinical case is related to a patient who is a carrier of the heterozygous c.1260+1del *LZTR1* variant, with a high probability of its negative dominant effect. As a result, the interaction of an MT protein with a WT protein partially inhibits the latter. This leads to a disruption of RAS proteins’ ubiquitination process, which results in the overactivation of the RAS/MAPK pathway and promotes excessive cell proliferation. In adults, the LZTR1 protein is expressed mainly in vascular tissue and selected types of hemopoietic cells. *LZTR1* is a tumor suppressor gene and negative regulator of the RAS/MAPK pathway. The heterogenous mutational score in *LZTR1* and its varied frequency are related to the poor specification of its impact on oncogenesis. Additionally, the reports of different treatment susceptibilities for carriers of different mutation variants in the *LZTR1* gene confirmed its high phenotypic heterogeneity and point to the need of segregation of discovered genetic variants according to their clinical significance. The patients’ history revealed cases of lung cancer and esophageal and kidney cancer in the paternal line. The maternal line manifested ovarian and colorectal cancer cases. Those data are comparable with already reported oncogenic variants of *LZTR1* gene. The reported c.1260+1del variant was described only in three heterozygotes. The study analyses, together with already reported data, determined the potentially significant impact of the described *LZTR1* variant in cancer development. The significance of *LZTR1* mutation in breast cancer susceptibility was confirmed in exome sequencing metanalysis. Increased risk of breast cancer was confirmed for *LZTR1* and four other genes, classified as tumor suppressor genes. Additionally, protein-truncated variants were identified for the *LZTR1, ATRIP*, and *BARD1* genes [55]. The last one is a known predictor of breast cancer and is included in NGS panels for breast and ovarian cancers. The clinical course of the disease with rapid progression and treatment resistance may be related to altered levels of RAS kinases, which may also promote the metastatic process. The resistance to hormonotherapy in described clinical cases may be possibly overcome by the use of MEK inhibitors, for example, trametinib. These types of therapeutics are dedicated for patients with overactivation of the MAPK-mediated pathway.

The conducted investigation confirmed that the *LZTR1*: c.1260+1del variant is a loss of function mutation, which is localized within Kelch’s motif. In this case there is a high probability of imatinib resistance, as was reported by Bigenzhan et al. However, an *in vitro* examination was performed for variants other than c.1260+1del in a homozygous form, while this case study represents a heterozygous patient with a germline mutation in *LZTR1*. The *in silico* analysis of *LZTR1:* c.1260+1del demonstrates a high possibility of tumorigenesis induced by this variant. These findings highlight the necessity of carefully monitoring patients with this variant. Moreover, patients with *LZTR1:* c.1260+1del could require changes in the recommended treatment strategies. It should be emphasized that it is necessary to verify the results of the presented analysis by *in vitro* and *in vivo* studies. Further functional studies in clinical models are urgently needed. Additional epidemiological studies on *LZTR1*-related phenotypes in oncological patients are required.

## 4. Materials and Methods

### 4.1. Molecular Diagnostics

In order to confirm the hereditary basis of the neoplastic disease (bilateral breast cancer in young patient), a panel study was performed. The study was approved by the Bioethical Committee, Medical University of Lublin, Poland, on 19 September 2024 (Approval code: KB-0024/130/09/2024). After obtaining informed consent for genetic testing, patients’ DNA was isolated from peripheral blood lymphocytes. The test was performed by means of the NGS technique using the panel of genes, the defects of which may correlate with an increased risk of malignant tumors, including breast cancer.

List of genes analyzed in the study (gene symbol according to HGVS; percentage of coverage): 30×/10×/5× for each gene: AIP 100; ALK 100; APC 100; ATM 100; AXIN2 100; BAP1 100; BARD1 100; BLM 100; BMPR1A 100; BRAF 99/100; BRIP1 100; CDC73 100; CDH1 100; CDK4 100; CDKN1B 100; CDKN2A 100; CEBPA 100; CHEK2 100; CXCR4 100; DDB2 100; DICER1 100; EPCAM 100; FANCC 100; FH 100; FLCN 100; GALNT12 100; GREM1 100; HNF1A 100; HOXB13 100; HRAS 100; KRAS 100; LZTR1 100; MAX 100; MEN1 94/96/99(); MET 100; MITF 100; MLH1 100; MLH3 100; MRE11 99/100; MSH2 100; MSH3 100; MSH6 100; MUTYH 100; NBN 100; NF1 100; NF2 100; NOD2 100; NTHL1 100; PALB2 100; PMS1 100; PMS2 100; POLD1 100; POLE 100; POT1 100; PRF1 100; PRKAR1A 100; PRSS1 100; PTCH1 100; PTEN 100; RAD50 100; RAD51C 100; RAD51D 100; RB1 96/99/99(); RET 100; SDHA 100; SDHAF2 100; SDHB 100; SDHC 100; SDHD 98/100; SMAD4 100; SMARCB1 100; STK11 100; TMEM127 100; TP53 100; TSC1 100; TSC2 100; VHL 100; WT1 100; XRCC2 100. Separate tests including the analysis of the BRCA1 and BRCA2 genes were performed.

NGS analysis was performed using Illumina technology. The Sureselect XT kit (Agilent) was used for library preparation. The whole library covered 10× > 99%, 30× > 98%. Rare variants in exons along with surrounding fragments of intronic sequences were analyzed. Variants found in the selected genes were filtered for their frequency in the general population, based on dbSNP and GnomAD and assuming a cutoff value of 1%. For bioinformatical analysis, the GRCh38 genome assembly was used. NGS analysis and sequencing result interpretation were performed in Medgen laboratory, Warsaw, Poland.

### 4.2. Bioinformatical Analysis Was Performed with the Use of Available Datasets and Bioinformatical Tools

The bioinformatical pipeline for the performed analysis is described in Table 4.

#### Analysis of Gene and Transcripts Structures Combined with *LZTR1:* c.1260+1del Pathogenicity Assessment

Analysis of the gene structure and localization of the c.1260+1del mutation were performed based on the data available in the Genome Aggregation Database (gnomAD), Ensembl genome browser, and GenBank databases (accessed 27 June 2025). We used the GRCh38 genome version, the same one used for the NGS result analysis. Sequences used for further analyses were extracted from the abovementioned databases. Nucleotide sequences are described Supplementary Materials File S1.

Firstly, in the study analyses, we used the consensus coding sequence (ccds) from Ensembl (CCDS33606.1) of wild-type (WT) *LZTR1* and a mutated *LZTR1* (MT) sequence was generated, consistent with c.1260+1del (the deletion of Guanine-residue in position 1260 of the coding sequence). The next step was to examine the position of this mutation within the gene’s open reading frame (ORF). Based on the position number from gnomAD, the position of *LZTR1:* c.1260+1del was matched in the whole genome assembly available in NCBI server. The mRNA sequence for the *LZTR1* gene was longer from the ccds record.

To predict molecular consequences of the *LZTR1* gene variant, we used the Variant Effect Predictor tool from the Expasy website [31]. Nevertheless, to predict the biological impact of the examined genetic variant, prediction of the ORF region was performed. Open reading frames in the described variant of *LZTR1* were analyzed with ORF Finder and GENESCAN [56,57].

### 4.3. Analysis of Genetic Variant Influence on Splicing

Gene variant influence on splicing was examined with HSF GenOmnis and a web-based application—MutationTaster results from MutationTaster [58,59]. Full result description from MutationTaster are available through the link in the citation [60]. Because of the variant mutation status (splice donor variant) and variant influence on splicing, BDGP: Splice Site Prediction by Neural Network was used to predict splice sites [61].

### 4.4. Gene Variant Transcript Prediction, Protein Structure Design, and Evaluation

The known structure of LZTR1 protein was used as a template. All generated protein structures by the used tools (SWISS-MODEL, AlphaFold3, and I-TASSER) were comparable.

Mutations which span the splice sites could result in three ways of alternative splicing. The first is a possibility of a frameshift, the second is exon skipping (exon with deleted nucleotide in splice site), and the third involves the use of the new cryptic splice sites. Because of the usage of bioinformatic tools alone, we decided to analyze these three possible events of splicing. A template ccds sequence from Ensemble (CCDS33606.1) was used. The ccds sequences of the determined splicing variant are presented in Supplementary Materials File S3.

First, the translation of wild-type (WT) *LZTR1* transcripts and mutated type (c.1260+1del variant) transcript were performed in the ExPASy Translational Tool [62,63]. The translation of WT sequences and its comparison with reference protein sequence in Clustul Omega were performed for tool evaluation.

The reported variant of the LZTR1 protein was tested with Expasy tool -SWISS-MODEL software (v101.0) [64,65]. Additional tools used for tertiary protein structure prediction were I-TAASER (MIT) and AlphaFold3.

The amino acid sequences were determined by the ORF Finder tool. Designated amino acid sequences were used as targets for protein structure prediction. For that, Expasy tool—SWISS-MODEL software (v101.0)was used. Predicted protein structures were evaluated by the ERRAT tool to check the quality factors of structures [66]. The evaluation of built protein structures showed a high quality score for every structure.

Predicted protein sequences of WT, MT1, MT2, MT3, and MT4 were analyzed by the InterPro tool to classify them to the protein families and predict the protein domains [67]. Moreover, to distinguish protein domains the Prosite tool and Smart tool were additionally used [68,69].

### 4.5. Analysis of Gene-Related Phenotypes

To investigate already reported data about the clinical significance of different mutation variants of the *LZTR1* gene, we used software from gnomAD (v2.1.1) (accessed on 12 March 2025) ClinVar (accessed on 14 March 2025), and Orphanet (accessed on 15 March 2025).

### 4.6. Analysis of Protein–Protein Interactions

Protein–protein interactions were analyzed using the STRING database [70]. The STRING database enabled us to find the known protein interactions and predicted interactions. Moreover, the Genemania tool was used for protein–protein interaction analysis (39). The Genemania tool helps with predicting gene function and their association network.

### 4.7. Protein Homology Analysis

LZTR1 protein homology was examined with the COBALT tool [71]. Homology examination was performed to determine multispecies changes in amino acid sequence and its alteration of conserved amino acids of LZTR1.

### 4.8. Model Organism’s Phenotype Analysis

Model organisms with a high *LZTR1* homology score were selected for phenotype examination. The ZFIN database marked two phenotypes related with the *LZTR1* gene [40]. Analysis of related phenotypes was performed in Alliancegenome.

## 5. Conclusions

The results of the *in silico* analysis of the *LZTR1:* c.1260+1del variant, in the context of the available literature data, indicates the high probability that this variant influences cancer development. Patients who are carriers of this variant may require a specialized therapeutic approach. Further *in vitro* and *in vivo* analyses are necessary to confirm the presented preliminary results. Each *in silico* examination should be confirmed by functional tests that measure transcriptomic and proteomic levels. The presented study did not include RNA evaluation. A proper investigation of *LZTR1* transcripts and LZTR1 protein levels in affected cells should be carried out. Hence, the obtained results are putative. Future studies utilizing an *in vitro* model will be used for the further examination and confirmation of the established hypothesis.

## Figures and Tables

**Table 1:** New donor spice sites predicted for *LZTR1* c.1260+1del by HSF GenOmnis and MutationTaster.

HSF Gen Omnis	Mutation Taster
GTTCCAGTTCTC	GTGTTTGG
GGTTCCATTTCT	TTTGGGGG
TGTGGGGCCTGT	TGGGGTGC

**Table 3:** Presentation of phenotypes related to the *LZTR1* gene variant reported on three databases.

Database	Reported Phenotypes Related with *LZTR1*
OMIM	Schwannomatosis 2, Noonan syndrome 2, Noonan syndrome 10
ClinVar	Noonan syndrome 10, Noonan syndrome 2, DiGeorge syndrome, epilepsy, intellectual disability, hydronephrosis, neurodevelopmental delay, global developmental delay, inborn genetic diseases, cat eye syndrome, aganglionic megacolon, rasopathy, inherited immunodeficiency diseases, congenital diaphragmatic hernia, oppositional defiant disorder, 22q11.2 central duplication syndrome, 22q11.2 central deletion syndrome, chromosome 22q11.2 microduplication syndrome, velocardiofacial syndrome, neurodevelopmental disorder, bladder exstrophy, schizophrenia, autistic disorder, cognitive impairment, ear malformation, VATER association, autism spectrum disorder, premature ovarian failure
Orphanet	Familial isolated café-au-lait macules, full schwannomatosis, giant cell glioblastoma, glioblastoma, gliosarcoma, Noonan syndrome

**Table 4:** Bioinformatics pipeline.

Tool	Latest Version/Platform Info	Thresholds/Notes
gnomAD	v4.1 (2024) and v2.1.1 (legacy)	LOEUF threshold: <0.6 (v4.1), <0.35 (v2.1.1); GroupMax FAF used for BA1/BS1 variant filtering
SnapGene Viewer	v8.1.0 (2025)	No specific thresholds; version compatibility: Windows 10+ (64-bit), macOS 10.14+
Variant Effect Predictor (VEP)	v112 and v113.3 (Ensembl)	No fixed thresholds; plugin-dependent; supports GRCh37 and GRCh38
ORF Finder	Web version (NCBI) and standalone Linux version	Default min ORF length: 75–300 nt; start codon: ATG or alternatives; nested ORFs optional
GENSCAN	Web server (MIT); original version from 1997	No numeric thresholds; organism-specific models; supports sequences up to 1 Mbp
HSF GenOmnis	https://hsf.genomnis.com/, accessed on 10 October 2024	Splice site impact: MaxEntScan + HSF matrix; ESE/ESS prediction: sensitivity ~0.83, specificity ~0.81
MutationTaster	MutationTaster2021 (GRCh37)	No fixed thresholds; integrates gnomAD, ExAC, and splice prediction; scores are probabilistic
SpliceAI	v1.3.1 (Illumina); also used via Broad’s SpliceAI Lookup	Recommended delta score threshold: ≥0.5 (high confidence), ≥0.2 (high recall); max distance: 50 bp
BDGP Splice Site Prediction	NNSPLICE v0.9 (1997)	Score threshold: typically ≥0.4–0.6 for reliable splice site prediction
SWISS-MODEL	Web-based (2025); version 101.0 of InterPro integration	No thresholds; uses QMEAN and LDDT for model quality; AlphaFold templates integrated
Prosite	Release 2025_03 (June 2025)	No thresholds; pattern/profile-based domain detection; integrated with InterPro
Smart	Integrated in InterPro (2025)	No thresholds; domain detection via HMMs
InterPro	Version 101.0 (2025)	No thresholds; integrates 13 databases including Pfam, SMART, PROSITE, etc.
STRING	Version 12.0 (2025)	Confidence score thresholds: low (0.15), medium (0.4), high (0.7), highest (0.9)
GenMANIA	v3.4.0Web-based; Cytoscape plugin available	No thresholds; uses label propagation and network weighting for gene function prediction
PipeAlign2	Web-based (LBGI); updated version of PipeAlign	No thresholds; used for MACS alignment and subfamily clustering

## Data Availability

Links to the datasets used and the results of the analysis performed are available in the citations. The sequences used for the analysis are described in the Supplementary Materials.

## Supplementary Materials

The following supporting information can be downloaded at: https://www.mdpi.com/article/10.3390/ijms26146704/s1.

## Abbreviations

The following abbreviations are used in this manuscript:

**ASCO**	American Society of Clinical Oncology
**ESMO**	European Society of Medical Oncology
***BRCA1/2***	*breast cancer gene*
**LZTR1**	leucine zipper-like transcriptional regulator 1
**NCCN**	National Comprehensive Cancer Network
**HER-2**	human epidermal growth factor receptor 2
**ER**	estrogen receptor
**PR**	progesterone receptor
***TP53***	tumor protein 53 gene
***PTEN***	tensin homology gene
***CDH1***	cadherin1 gene
***STK11***	serine/threonine kinase 11 gene
***ATM***	ataxia-telangiectasia mutated gene
***BARD1***	BRCA1-associated RING domain 1gene
***CHECK2***	checkpoint kinase 2
***PALB*2**	partner and localizer of BRCA2 gene
***RAD51 gene***	adiation sensitive protein 51
**Ki-67**	protein marker of cell proliferation
**NOS**	no otherwise specified according to the WHO 2018 classification
**CDK4/6**	cyclin-dependent kinase 4 and 6.
***AIP***	aryl hydrocarbon receptor-interacting gene.
***ALK***	anaplastic lymphoma kinase gene
***ATM***	ataxia telangiectasia mutated
***AXIN2***	axis inhibition protein 2
***BAP1***	BRCA1-associated protein 1
***BARD1***	BRCA1-associated RING domain 1
***BLM***	Bloom syndrome RecQ-like helicase
***BMPR1A***	bone morphogenetic protein receptor type 1A
***BRAF***	B-Raf proto-oncogene, serine/threonine kinase
***BRIP1***	BRCA1 interacting protein C-terminal helicase 1
***CDC73***	cell division cycle 73
***CDH1***	cadherin 1
***CDK4***	cyclin-dependent kinase 4
***CDKN1B***	cyclin-dependent kinase inhibitor 1B
***CDKN2A***	cyclin-dependent kinase inhibitor 2A
***CEBPA***	CCAAT enhancer binding protein alpha
***CHEK2***	checkpoint kinase 2
***CXCR4***	C-X-C motif chemokine receptor 4
***DDB2***	damage specific DNA binding protein 2
***DICER1***	dicer 1, ribonuclease III
***EPCAM***	epithelial cell adhesion molecule
***FANCC***	Fanconi anemia complementation group C
***FH***	fumarate hydratase
***FLCN***	folliculin
***GALNT12***	polypeptide N-acetylgalactosaminyltransferase 12
***GREM1***	gremlin 1, DAN family BMP antagonist
***HNF1A***	hepatocyte nuclear factor 1 alpha
***HOXB13***	homeobox B13
***HRAS***	Harvey rat sarcoma viral oncogene homolog
***KRAS***	Kirsten rat sarcoma viral oncogene homolog
***LZTR1***	leucine zipper-like transcription regulator 1
***MAX***	MYC-associated factor X
***MEN1***	multiple endocrine neoplasia type 1
***MET***	MET proto-oncogene, receptor tyrosine kinase
***MITF***	Microphthalmia-associated transcription factor
***MLH1***	MutL homolog 1
***MLH3***	MutL homolog 3
***MRE11***	MRE11 homolog, double-strand break repair nuclease
***MSH2***	MutS homolog 2
***MSH3***	MutS homolog 3
***MSH6***	MutS homolog 6
***MUTYH***	MutY DNA glycosylase
***NBN***	nibrin
***NF1***	neurofibromin 1
***NF2***	neurofibromin 2
***NOD2***	nucleotide-binding oligomerization domain containing 2
***NTHL*1**	endonuclease III-like 1
***PALB2***	partner and localizer of BRCA2
***PMS1***	PMS1 homolog 1, mismatch repair system component
***PMS2***	PMS2 homolog 2, mismatch repair system component
***POLD1***	DNA polymerase delta 1
***POLE***	DNA polymerase epsilon
***POT1***	protection of telomeres 1
***PRF1***	perforin 1
***PRKAR1A***	protein kinase CAMP-dependent regulatory subunit alpha
***PRSS1***	protease, serine 1
***PTCH1***	patched 1
***PTEN***	phosphatase and tensin homolog
***RAD50***	RAD50 double-strand break repair protein
***RAD51C***	RAD51 paralog C
***RAD51D***	RAD51 paralog D
***RB1***	retinoblastoma 1
***RET***	RET proto-oncogene
***SDHA***	succinate dehydrogenase complex flavoprotein subunit A
***SDHAF2***	succinate dehydrogenase complex assembly factor 2
***SDHB***	succinate dehydrogenase complex iron sulfur subunit B
***SDHC***	succinate dehydrogenase complex subunit C
***SDHD***	succinate dehydrogenase complex subunit D
***SMAD4***	SMAD family member 4
***SMARCB1***	SWI/SNF-related matrix-associated actin-dependent regulator of chromatin subfamily B member 1
***STK11***	serine/threonine kinase 11
***TMEM127***	transmembrane protein 127
***TP53***	tumor protein P53
***TSC1***	tuberous sclerosis complex 1
***TSC2***	tuberous sclerosis complex 2
***VHL***	Von Hippel–Lindau tumor suppressor
***WT1***	Wilms tumor 1
***XRCC2***	X-ray repair cross complementing 2
**NGS**	next-generation sequencing
**dsSNP**	Database of Single Nucleotide Polymorphisms
**GRCh38**	Genome Reference Consortium Human Build 38
**GnomAD**	Genome Aggregation Database
**BTB**	broad-complex, tramtrack, and bric-a-brac
**BACK**	BTB and C-terminal Kelch
**Kelch**	Kelch repeat domain
**E3**	E3 ubiquitin ligase
**CUL3**	cullin 3
**ORF**	open reading frame
**WT**	wild type
**MT1/2/3/4**	mutation variant1/2/3/4
**ccds**	consensus coding sequence
**NCBI**	National Center for Biotechnology Information
**MIT**	Massachusetts Institute of Technology
**RAT**	Rattus norvegicus
**MOUSE**	Mus musculus
**DANRE**	Danio rerio
**CHIC**	Gallus gallus
**XENTR**	Xenopus tropicalis
**ZFIN database**	Zebrafish Model Organism Database
**RAS**	rat sarcoma viral oncogene homolog
**MAPK**	mitogen-activated protein kinase
**MEK**	MAPK/ERK kinase (mitogen-activated protein kinase/extracellular signal-regulated kinase)
