# Supplementary material for: LZTR1: c.1260+1del Variant as a Significant Predictor of Early-Age Breast Cancer Development: Case Report Combined with In Silico Analysis
Source: Int J Mol Sci. 2025 Jul 12;26(14):6704. doi: 10.3390/ijms26146704 (PMC12294705; doi:10.3390/ijms26146704)
Supplement: Supplementary file 1 [file ijms-26-06704-s001.zip › supplementary S1 (LZTR1 case study).pdf]

>CCDS33606.1 WT LZTR1
ATGGCTGGACCGGGCAGCACGGGGGGGCAGATCGGGGCTGCGGCCCTGGCAGGCGGCGCGCGGTCCAAGG
TAGCCCCGAGCGTGGACTTCGACCATAGCTGCTCGGACAGTGTCGAGTACCTGACGCTCAACTTCGGGCC
CTTCGAAACAGTGCATCGCTGGCGGCGCCTCCCGCCCTGCGACGAGTTCGTGGGTGCCCGGCGCAGCAAG
CACACAGTGGTGGCCTATAAAGATGCCATTTATGTATTTGGTGGAGACAATGGGAAGACCATGCTCAATG
ACCTCCTGCGGTTCGATGTGAAAGACTGCTCCTGGTGCAGGGCCTTTACCACTGGGACCCCACCGGCCCC
CCGTTACCACCACTCGGCCGTCGTCTATGGGAGCAGCATGTTTGTCTTTGGGGGTTACACTGGGGACATT
TATTCCAATTCTAACTTGAAGAATAAAAACGACCTCTTTGAATACAAGTTTGCAACTGGCCAGTGGACGG
AGTGGAAAATTGAAGGACGGTTGCCAGTCGCTAGGTCAGCCCATGGGGCCACGGTGTACAGTGACAAGCT
GTGGATCTTTGCTGGCTATGACGGCAACGCCAGGTTGAATGACATGTGGACAATTGGCCTCCAGGACCGA
GAGCTCACCTGCTGGGAGGAGGTGGCCCAGAGTGGCGAGATCCCCCCATCTTGCTGCAACTTCCCCGTGG
CTGTGTGCCGGGACAAGATGTTTGTATTCTCTGGGCAAAGCGGAGCCAAAATAACCAACAACCTCTTCCA
GTTTGAATTCAAGGACAAGACGTGGACACGCATCCCAACTGAACACCTGCTCCGGGGCTCCCCACCACCC
CCGCAGCGGCGCTACGGGCATACCATGGTGGCCTTTGACCGCCACCTCTATGTGTTTGGGGGTGCGGCCG
ACAACACGCTGCCCAACGAGCTGCACTGCTATGACGTGGACTTCCAGACCTGGGAGGTCGTCCAGCCCAG
CTCCGACAGCGAGGTTGGTGGGGCTGAAGTGCCCGAGCGAGCCTGTGCTTCCGAGGAGGTGCCCACCCTG
ACCTATGAGGAGCGGGTTGGCTTCAAGAAGTCCCGAGATGTGTTTGGCCTGGACTTTGGCACCACCTCAG
CCAAGCAGCCCACCCAGCCTGCCTCGGAGCTGCCCAGTGGGAGGCTCTTCCACGCGGCTGCTGTCATCTC
GGACGCCATGTACATCTTCGGGGGCACGGTGGACAACAACATCCGCAGCGGGGAGATGTACAGGTTCCAG
TTCTCCTGTTACCCTAAATGCACGCTGCACGAGGACTACGGGCGGCTGTGGGAGAGCCGCCAGTTCTGCG
ACGTGGAGTTCGTGCTGGGTGAGAAGGAGGAGTGCGTGCAGGGCCACGTAGCCATTGTCACAGCGCGGAG
CCGCTGGCTTCGCAGGAAGATCACGCAGGCGCGGGAGAGGCTGGCCCAGAAGCTGGAGCAGGAGGCCGCC
CCAGTTCCCAGGGAGGCCCCCGGCGTGGCTGCTGGTGGGGCCCGGCCGCCCCTGCTGCACGTGGCCATCC
GGGAGGCCGAGGCCCGGCCCTTCGAGGTGCTCATGCAGTTCCTCTACACCGACAAGATCAAATACCCACG
GAAAGGCCATGTGGAGGATGTGCTGCTCATCATGGATGTGTACAAACTGGCACTGAGCTTCCAGTTGTGC
CGCCTGGAGCAGCTGTGCCGCCAGTACATCGAGGCCTCCGTGGACCTGCAGAACGTGCTGGTTGTGTGCG
AGAGTGCCGCCCGGCTGCAGCTGAGCCAACTCAAGGAGCACTGCCTGAACTTCGTGGTAAAGGAGTCCCA
CTTCAACCAGGTGATCATGATGAAGGAGTTCGAGCGCCTCTCCTCTCCACTGATAGTGGAGATTGTGCGG
CGGAAGCAGCAGCCGCCCCCTCGCACTCCCTTGGACCAGCCAGTGGACATTGGCACATCTCTGATCCAGG
ACATGAAGGCATACCTGGAGGGAGCGGGCGCGGAATTCTGTGACATCACTCTGTTGCTTGACGGGCACCC
ACGGCCAGCCCACAAGGCTATCCTGGCCGCCCGCTCCAGCTACTTTGAAGCCATGTTCCGGTCCTTCATG
CCCGAAGATGGGCAGGTGAACATCTCCATCGGGGAGATGGTGCCCAGCAGGCAGGCCTTCGAGTCCATGC
TGCGCTACATCTACTACGGCGAGGTCAACATGCCGCCCGAGGACTCGCTCTACTTGTTTGCGGCCCCCTA
CTACTACGGCTTCTACAACAACCGGCTGCAGGCGTACTGCAAGCAGAACCTGGAGATGAACGTGACGGTG
CAGAACGTGCTGCAGATCCTGGAGGCAGCTGACAAAACGCAGGCACTGGACATGAAGCGGCACTGCCTGC
ACATCATTGTGCACCAGTTCACCAAGGTCTCCAAGTTGCCCACCCTGCGGTCGCTGAGCCAGCAGCTGCT
GCTGGACATCATAGACTCCCTGGCCTCCCACATCTCAGACAAGCAGTGCGCAGAGCTGGGCGCCGACATC
TGA

>LZTR1: ccds for c.1260+1del
ATGGCTGGACCGGGCAGCACGGGGGGGCAGATCGGGGCTGCGGCCCTGGCAGGCGGCGCGCGGTCCAAGG
TAGCCCCGAGCGTGGACTTCGACCATAGCTGCTCGGACAGTGTCGAGTACCTGACGCTCAACTTCGGGCC
CTTCGAAACAGTGCATCGCTGGCGGCGCCTCCCGCCCTGCGACGAGTTCGTGGGTGCCCGGCGCAGCAAG
CACACAGTGGTGGCCTATAAAGATGCCATTTATGTATTTGGTGGAGACAATGGGAAGACCATGCTCAATG
ACCTCCTGCGGTTCGATGTGAAAGACTGCTCCTGGTGCAGGGCCTTTACCACTGGGACCCCACCGGCCCC
CCGTTACCACCACTCGGCCGTCGTCTATGGGAGCAGCATGTTTGTCTTTGGGGGTTACACTGGGGACATT
TATTCCAATTCTAACTTGAAGAATAAAAACGACCTCTTTGAATACAAGTTTGCAACTGGCCAGTGGACGG
AGTGGAAAATTGAAGGACGGTTGCCAGTCGCTAGGTCAGCCCATGGGGCCACGGTGTACAGTGACAAGCT
GTGGATCTTTGCTGGCTATGACGGCAACGCCAGGTTGAATGACATGTGGACAATTGGCCTCCAGGACCGA
GAGCTCACCTGCTGGGAGGAGGTGGCCCAGAGTGGCGAGATCCCCCCATCTTGCTGCAACTTCCCCGTGG
CTGTGTGCCGGGACAAGATGTTTGTATTCTCTGGGCAAAGCGGAGCCAAAATAACCAACAACCTCTTCCA
GTTTGAATTCAAGGACAAGACGTGGACACGCATCCCAACTGAACACCTGCTCCGGGGCTCCCCACCACCC
CCGCAGCGGCGCTACGGGCATACCATGGTGGCCTTTGACCGCCACCTCTATGTGTTTGGGGGTGCGGCCG
ACAACACGCTGCCCAACGAGCTGCACTGCTATGACGTGGACTTCCAGACCTGGGAGGTCGTCCAGCCCAG
CTCCGACAGCGAGGTTGGTGGGGCTGAAGTGCCCGAGCGAGCCTGTGCTTCCGAGGAGGTGCCCACCCTG
ACCTATGAGGAGCGGGTTGGCTTCAAGAAGTCCCGAGATGTGTTTGGCCTGGACTTTGGCACCACCTCAG
CCAAGCAGCCCACCCAGCCTGCCTCGGAGCTGCCCAGTGGGAGGCTCTTCCACGCGGCTGCTGTCATCTC
GGACGCCATGTACATCTTCGGGGGCACGGTGGACAACAACATCCGCAGCGGGGAGATGTACAGGTTCCAT
TCTCCTGTTACCCTAAATGCACGCTGCACGAGGACTACGGGCGGCTGTGGGAGAGCCGCCAGTTCTGCGA
CGTGGAGTTCGTGCTGGGTGAGAAGGAGGAGTGCGTGCAGGGCCACGTAGCCATTGTCACAGCGCGGAGC
CGCTGGCTTCGCAGGAAGATCACGCAGGCGCGGGAGAGGCTGGCCCAGAAGCTGGAGCAGGAGGCCGCCC

CAGTTCCCAGGGAGGCCCCCGGCGTGGCTGCTGGTGGGGCCCGGCCGCCCCTGCTGCACGTGGCCATCCG
GGAGGCCGAGGCCCGGCCCTTCGAGGTGCTCATGCAGTTCCTCTACACCGACAAGATCAAATACCCACGG
AAAGGCCATGTGGAGGATGTGCTGCTCATCATGGATGTGTACAAACTGGCACTGAGCTTCCAGTTGTGCC
GCCTGGAGCAGCTGTGCCGCCAGTACATCGAGGCCTCCGTGGACCTGCAGAACGTGCTGGTTGTGTGCGA
GAGTGCCGCCCGGCTGCAGCTGAGCCAACTCAAGGAGCACTGCCTGAACTTCGTGGTAAAGGAGTCCCAC
TTCAACCAGGTGATCATGATGAAGGAGTTCGAGCGCCTCTCCTCTCCACTGATAGTGGAGATTGTGCGGC
GGAAGCAGCAGCCGCCCCCTCGCACTCCCTTGGACCAGCCAGTGGACATTGGCACATCTCTGATCCAGGA
CATGAAGGCATACCTGGAGGGAGCGGGCGCGGAATTCTGTGACATCACTCTGTTGCTTGACGGGCACCCA
CGGCCAGCCCACAAGGCTATCCTGGCCGCCCGCTCCAGCTACTTTGAAGCCATGTTCCGGTCCTTCATGC
CCGAAGATGGGCAGGTGAACATCTCCATCGGGGAGATGGTGCCCAGCAGGCAGGCCTTCGAGTCCATGCT
GCGCTACATCTACTACGGCGAGGTCAACATGCCGCCCGAGGACTCGCTCTACTTGTTTGCGGCCCCCTAC
TACTACGGCTTCTACAACAACCGGCTGCAGGCGTACTGCAAGCAGAACCTGGAGATGAACGTGACGGTGC
AGAACGTGCTGCAGATCCTGGAGGCAGCTGACAAAACGCAGGCACTGGACATGAAGCGGCACTGCCTGCA
CATCATTGTGCACCAGTTCACCAAGGTCTCCAAGTTGCCCACCCTGCGGTCGCTGAGCCAGCAGCTGCTG
CTGGACATCATAGACTCCCTGGCCTCCCACATCTCAGACAAGCAGTGCGCAGAGCTGGGCGCCGACATCT
GA

> Exon 11 from NCBI Reference Sequence: NG_034193.1
CTGCCCAGTGGGAGGCTCTTCCACGCGGCTGCTGTCATCTCGGACGCCATGTACATCTTCGGGGGCACGG
TGGACAACAACATCCGCAGCGGGGAGATGTACAGGTTCCAG
