# Supplementary material for: LZTR1: c.1260+1del Variant as a Significant Predictor of Early-Age Breast Cancer Development: Case Report Combined with In Silico Analysis
Source: Int J Mol Sci. 2025 Jul 12;26(14):6704. doi: 10.3390/ijms26146704 (PMC12294705; doi:10.3390/ijms26146704)
Supplement: Supplementary file 1 [file ijms-26-06704-s001.zip › supplementary S2 (LZTR1 case study).pdf]

	**WT Sequences**
**Donor splice site in exon 11th or its region**	GTTCCAGTTCTC
**CDS**	ATGGCTGGACCGGGCAGCACGGGGGGGCAGATCGGGGCTGCGGCCCTGGCAGGCGGCGCGCGGTCCAAGGTAGCCCCGAGCGTGGACTTCGACCATAGCTGCTCG<br>GACAGTGTCGAGTACCTGACGCTCAACTTCGGGCCCTTCGAAACAGTGCATCGCTGGCGGCGCCTCCCGCCCTGCGACGAGTTCGTGGGTGCCCGGCGCAGCAAGC<br>ACACAGTGGTGGCCTATAAAGATGCCATTTATGTATTTGGTGGAGACAATGGGAAGACCATGCTCAATGACCTCCTGCGGTTCGATGTGAAAGACTGCTCCTGGTG<br>CAGGGCCTTTACCACTGGGACCCCACCGGCCCCCCGTTACCACCACTCGGCCGTCGTCTATGGGAGCAGCATGTTTGTCTTTGGGGGTTACACTGGGGACATTTATT<br>CCAATTCTAACTTGAAGAATAAAAACGACCTCTTTGAATACAAGTTTGCAACTGGCCAGTGGACGGAGTGGAAAATTGAAGGACGGTTGCCAGTCGCTAGGTCAGC<br>CCATGGGGCCACGGTGTACAGTGACAAGCTGTGGATCTTTGCTGGCTATGACGGCAACGCCAGGTTGAATGACATGTGGACAATTGGCCTCCAGGACCGAGAGCTC<br>ACCTGCTGGGAGGAGGTGGCCCAGAGTGGCGAGATCCCCCCATCTTGCTGCAACTTCCCCGTGGCTGTGTGCCGGGACAAGATGTTTGTATTCTCTGGGCAAAGCG<br>GAGCCAAAATAACCAACAACCTCTTCCAGTTTGAATTCAAGGACAAGACGTGGACACGCATCCCAACTGAACACCTGCTCCGGGGCTCCCCACCACCCCCGCAGCG<br>GCGCTACGGGCATACCATGGTGGCCTTTGACCGCCACCTCTATGTGTTTGGGGGTGCGGCCGACAACACGCTGCCCAACGAGCTGCACTGCTATGACGTGGACTTCC<br>AGACCTGGGAGGTCGTCCAGCCCAGCTCCGACAGCGAGGTTGGTGGGGCTGAAGTGCCCGAGCGAGCCTGTGCTTCCGAGGAGGTGCCCACCCTGACCTATGAGG<br>AGCGGGTTGGCTTCAAGAAGTCCCGAGATGTGTTTGGCCTGGACTTTGGCACCACCTCAGCCAAGCAGCCCACCCAGCCTGCCTCGGAGCTGCCCAGTGGGAGGCT<br>CTTCCACGCGGCTGCTGTCATCTCGGACGCCATGTACATCTTCGGGGGCACGGTGGACAACAACATCCGCAGCGGGGAGATGTACAGGTTCCAGTTCTCCTGTTACC<br>CTAAATGCACGCTGCACGAGGACTACGGGCGGCTGTGGGAGAGCCGCCAGTTCTGCGACGTGGAGTTCGTGCTGGGTGAGAAGGAGGAGTGCGTGCAGGGCCACG<br>TAGCCATTGTCACAGCGCGGAGCCGCTGGCTTCGCAGGAAGATCACGCAGGCGCGGGAGAGGCTGGCCCAGAAGCTGGAGCAGGAGGCCGCCCCAGTTCCCAGGG<br>AGGCCCCCGGCGTGGCTGCTGGTGGGGCCCGGCCGCCCCTGCTGCACGTGGCCATCCGGGAGGCCGAGGCCCGGCCCTTCGAGGTGCTCATGCAGTTCCTCTACAC<br>CGACAAGATCAAATACCCACGGAAAGGCCATGTGGAGGATGTGCTGCTCATCATGGATGTGTACAAACTGGCACTGAGCTTCCAGTTGTGCCGCCTGGAGCAGCTG<br>TGCCGCCAGTACATCGAGGCCTCCGTGGACCTGCAGAACGTGCTGGTTGTGTGCGAGAGTGCCGCCCGGCTGCAGCTGAGCCAACTCAAGGAGCACTGCCTGAACT<br>TCGTGGTAAAGGAGTCCCACTTCAACCAGGTGATCATGATGAAGGAGTTCGAGCGCCTCTCCTCTCCACTGATAGTGGAGATTGTGCGGCGGAAGCAGCAGCCGCC<br>CCCTCGCACTCCCTTGGACCAGCCAGTGGACATTGGCACATCTCTGATCCAGGACATGAAGGCATACCTGGAGGGAGCGGGCGCGGAATTCTGTGACATCACTCTG<br>TTGCTTGACGGGCACCCACGGCCAGCCCACAAGGCTATCCTGGCCGCCCGCTCCAGCTACTTTGAAGCCATGTTCCGGTCCTTCATGCCCGAAGATGGGCAGGTGA<br>ACATCTCCATCGGGGAGATGGTGCCCAGCAGGCAGGCCTTCGAGTCCATGCTGCGCTACATCTACTACGGCGAGGTCAACATGCCGCCCGAGGACTCGCTCTACTT<br>GTTTGCGGCCCCCTACTACTACGGCTTCTACAACAACCGGCTGCAGGCGTACTGCAAGCAGAACCTGGAGATGAACGTGACGGTGCAGAACGTGCTGCAGATCCTG<br>GAGGCAGCTGACAAAACGCAGGCACTGGACATGAAGCGGCACTGCCTGCACATCATTGTGCACCAGTTCACCAAGGTCTCCAAGTTGCCCACCCTGCGGTCGCTGA<br>GCCAGCAGCTGCTGCTGGACATCATAGACTCCCTGGCCTCCCACATCTCAGACAAGCAGTGCGCAGAGCTGGGCGCCGACATCTGA
**Aminoacid**	MAGPGSTGGQIGAAALAGGARSKVAPSVDFDHSCSDSVEYLTLNFGPFETVHRWRRLPPCDEFVGARRSKHTVVAYKDAIYVFGGDNGKTMLNDLLRFDVKDCSWCRA<br>FTTGTPPAPRYHHSAVVYGSSMFVFGGYTGDIYSNSNLKNKNDLFEYKFATGQWTEWKIEGRLPVARSAHGATVYSDKLWIFAGYDGNARLNDMWTIGLQDRELTCWE<br>EVAQSGEIPPSCCNFPVAVCRDKMFVFSGQSGAKITNNLFQFEFKDKTWTRIPTEHLLRGSPPPPQRRYGHTMVAFDRHLYVFGGAADNTLPNELHCYDVDFQTWEVVQP<br>SSDSEVGGAEVPERACASEEVPTLTYEERVGFKKSRDVFGLDFGTTSAKQPTQPASELPSGRLFHAAAVISDAMYIFGGTVDNNIRSGEMYRFQFSCYPKCTLHEDYGRLW<br>ESRQFCDVEFVLGEKEECVQGHVAIVTARSRWLRRKITQARERLAQKLEQEAAPVPREAPGVAAGGARPPLLHVAIREAEARPFEVLMQFLYTDKIKYPRKGHVEDVLLI<br>MDVYKLALSFQLCRLEQLCRQYIEASVDLQNVLVVCESAARLQLSQLKEHCLNFVVKESHFNQVIMMKEFERLSSPLIVEIVRRKQQPPPRTPLDQPVDIGTSLIQDMKAYL<br>EGAGAEFCDITLLLDGHPRPAHKAILAARSSYFEAMFRSFMPEDGQVNISIGEMVPSRQAFESMLRYIYYGEVNMPPEDSLYLFAAPYYYGFYNNRLQAYCKQNLEMNVT<br>VQNVLQILEAADKTQALDMKRHCLHIIVHQFTKVSKLPTLRSLSQQLLLDIIDSLASHISDKQCAELGADI

	MT1 Sequences
**Donor splice site in exon 11th or its region**	Exon skipping
**CDS**	ATGGCTGGACCGGGCAGCACGGGGGGGCAGATCGGGGCTGCGGCCCTGGCAGGCGGCGCGCGGTCCAAGGTAGCCCCGAGCGTGGACTTCGACCATAGCTGCTCG<br>GACAGTGTCGAGTACCTGACGCTCAACTTCGGGCCCTTCGAAACAGTGCATCGCTGGCGGCGCCTCCCGCCCTGCGACGAGTTCGTGGGTGCCCGGCGCAGCAAGC<br>ACACAGTGGTGGCCTATAAAGATGCCATTTATGTATTTGGTGGAGACAATGGGAAGACCATGCTCAATGACCTCCTGCGGTTCGATGTGAAAGACTGCTCCTGGTG<br>CAGGGCCTTTACCACTGGGACCCCACCGGCCCCCCGTTACCACCACTCGGCCGTCGTCTATGGGAGCAGCATGTTTGTCTTTGGGGGTTACACTGGGGACATTTATT<br>CCAATTCTAACTTGAAGAATAAAAACGACCTCTTTGAATACAAGTTTGCAACTGGCCAGTGGACGGAGTGGAAAATTGAAGGACGGTTGCCAGTCGCTAGGTCAGC<br>CCATGGGGCCACGGTGTACAGTGACAAGCTGTGGATCTTTGCTGGCTATGACGGCAACGCCAGGTTGAATGACATGTGGACAATTGGCCTCCAGGACCGAGAGCTC<br>ACCTGCTGGGAGGAGGTGGCCCAGAGTGGCGAGATCCCCCCATCTTGCTGCAACTTCCCCGTGGCTGTGTGCCGGGACAAGATGTTTGTATTCTCTGGGCAAAGCG<br>GAGCCAAAATAACCAACAACCTCTTCCAGTTTGAATTCAAGGACAAGACGTGGACACGCATCCCAACTGAACACCTGCTCCGGGGCTCCCCACCACCCCCGCAGCG<br>GCGCTACGGGCATACCATGGTGGCCTTTGACCGCCACCTCTATGTGTTTGGGGGTGCGGCCGACAACACGCTGCCCAACGAGCTGCACTGCTATGACGTGGACTTCC<br>AGACCTGGGAGGTCGTCCAGCCCAGCTCCGACAGCGAGGTTGGTGGGGCTGAAGTGCCCGAGCGAGCCTGTGCTTCCGAGGAGGTGCCCACCCTGACCTATGAGG<br>AGCGGGTTGGCTTCAAGAAGTCCCGAGATGTGTTTGGCCTGGACTTTGGCACCACCTCAGCCAAGCAGCCCACCCAGCCTGCCTCGGAGTTCTCCTGTTACCCTAAA<br>TGCACGCTGCACGAGGACTACGGGCGGCTGTGGGAGAGCCGCCAGTTCTGCGACGTGGAGTTCGTGCTGGGTGAGAAGGAGGAGTGCGTGCAGGGCCACGTAGCC<br>ATTGTCACAGCGCGGAGCCGCTGGCTTCGCAGGAAGATCACGCAGGCGCGGGAGAGGCTGGCCCAGAAGCTGGAGCAGGAGGCCGCCCCAGTTCCCAGGGAGGCC<br>CCCGGCGTGGCTGCTGGTGGGGCCCGGCCGCCCCTGCTGCACGTGGCCATCCGGGAGGCCGAGGCCCGGCCCTTCGAGGTGCTCATGCAGTTCCTCTACACCGACA<br>AGATCAAATACCCACGGAAAGGCCATGTGGAGGATGTGCTGCTCATCATGGATGTGTACAAACTGGCACTGAGCTTCCAGTTGTGCCGCCTGGAGCAGCTGTGCCG<br>CCAGTACATCGAGGCCTCCGTGGACCTGCAGAACGTGCTGGTTGTGTGCGAGAGTGCCGCCCGGCTGCAGCTGAGCCAACTCAAGGAGCACTGCCTGAACTTCGTG<br>GTAAAGGAGTCCCACTTCAACCAGGTGATCATGATGAAGGAGTTCGAGCGCCTCTCCTCTCCACTGATAGTGGAGATTGTGCGGCGGAAGCAGCAGCCGCCCCCTC<br>GCACTCCCTTGGACCAGCCAGTGGACATTGGCACATCTCTGATCCAGGACATGAAGGCATACCTGGAGGGAGCGGGCGCGGAATTCTGTGACATCACTCTGTTGCT<br>TGACGGGCACCCACGGCCAGCCCACAAGGCTATCCTGGCCGCCCGCTCCAGCTACTTTGAAGCCATGTTCCGGTCCTTCATGCCCGAAGATGGGCAGGTGAACATC<br>TCCATCGGGGAGATGGTGCCCAGCAGGCAGGCCTTCGAGTCCATGCTGCGCTACATCTACTACGGCGAGGTCAACATGCCGCCCGAGGACTCGCTCTACTTGTTTGC<br>GGCCCCCTACTACTACGGCTTCTACAACAACCGGCTGCAGGCGTACTGCAAGCAGAACCTGGAGATGAACGTGACGGTGCAGAACGTGCTGCAGATCCTGGAGGC<br>AGCTGACAAAACGCAGGCACTGGACATGAAGCGGCACTGCCTGCACATCATTGTGCACCAGTTCACCAAGGTCTCCAAGTTGCCCACCCTGCGGTCGCTGAGCCAG<br>CAGCTGCTGCTGGACATCATAGACTCCCTGGCCTCCCACATCTCAGACAAGCAGTGCGCAGAGCTGGGCGCCGACATCTGA
**Aminoacid**	MAGPGSTGGQIGAAALAGGARSKVAPSVDFDHSCSDSVEYLTLNFGPFETVHRWRRLPPCDEFVGARRSKHTVVAYKDAIYVFGGDNGKTMLNDLLRFDVKDCSWCRA<br>FTTGTPPAPRYHHSAVVYGSSMFVFGGYTGDIYSNSNLKNKNDLFEYKFATGQWTEWKIEGRLPVARSAHGATVYSDKLWIFAGYDGNARLNDMWTIGLQDRELTCWE<br>EVAQSGEIPPSCCNFPVAVCRDKMFVFSGQSGAKITNNLFQFEFKDKTWTRIPTEHLLRGSPPPPQRRYGHTMVAFDRHLYVFGGAADNTLPNELHCYDVDFQTWEVVQP<br>SSDSEVGGAEVPERACASEEVPTLTYEERVGFKKSRDVFGLDFGTTSAKQPTQPASEFSCYPKCTLHEDYGRLWESRQFCDVEFVLGEKEECVQGHVAIVTARSRWLRRKI<br>TQARERLAQKLEQEAAPVPREAPGVAAGGARPPLLHVAIREAEARPFEVLMQFLYTDKIKYPRKGHVEDVLLIMDVYKLALSFQLCRLEQLCRQYIEASVDLQNVLVVCE<br>SAARLQLSQLKEHCLNFVVKESHFNQVIMMKEFERLSSPLIVEIVRRKQQPPPRTPLDQPVDIGTSLIQDMKAYLEGAGAEFCDITLLLDGHPRPAHKAILAARSSYFEAMFR<br>SFMPEDGQVNISIGEMVPSRQAFESMLRYIYYGEVNMPPEDSLYLFAAPYYYGFYNNRLQAYCKQNLEMNVTVQNVLQILEAADKTQALDMKRHCLHIIVHQFTKVSKLP<br>TLRSLSQQLLLDIIDSLASHISDKQCAELGADI

	MT2 Sequences
**Donor splice site in exon 11th or its region**	GTTCCAGTTCTC
**CDS**	ATGGCTGGACCGGGCAGCACGGGGGGGCAGATCGGGGCTGCGGCCCTGGCAGGCGGCGCGCGGTCCAAGGTAGCCCCGAGCGTGGACTTCGACCATAGCTGCTCG<br>GACAGTGTCGAGTACCTGACGCTCAACTTCGGGCCCTTCGAAACAGTGCATCGCTGGCGGCGCCTCCCGCCCTGCGACGAGTTCGTGGGTGCCCGGCGCAGCAAGC<br>ACACAGTGGTGGCCTATAAAGATGCCATTTATGTATTTGGTGGAGACAATGGGAAGACCATGCTCAATGACCTCCTGCGGTTCGATGTGAAAGACTGCTCCTGGTG<br>CAGGGCCTTTACCACTGGGACCCCACCGGCCCCCCGTTACCACCACTCGGCCGTCGTCTATGGGAGCAGCATGTTTGTCTTTGGGGGTTACACTGGGGACATTTATT<br>CCAATTCTAACTTGAAGAATAAAAACGACCTCTTTGAATACAAGTTTGCAACTGGCCAGTGGACGGAGTGGAAAATTGAAGGACGGTTGCCAGTCGCTAGGTCAGC<br>CCATGGGGCCACGGTGTACAGTGACAAGCTGTGGATCTTTGCTGGCTATGACGGCAACGCCAGGTTGAATGACATGTGGACAATTGGCCTCCAGGACCGAGAGCTC<br>ACCTGCTGGGAGGAGGTGGCCCAGAGTGGCGAGATCCCCCCATCTTGCTGCAACTTCCCCGTGGCTGTGTGCCGGGACAAGATGTTTGTATTCTCTGGGCAAAGCG<br>GAGCCAAAATAACCAACAACCTCTTCCAGTTTGAATTCAAGGACAAGACGTGGACACGCATCCCAACTGAACACCTGCTCCGGGGCTCCCCACCACCCCCGCAGCG<br>GCGCTACGGGCATACCATGGTGGCCTTTGACCGCCACCTCTATGTGTTTGGGGGTGCGGCCGACAACACGCTGCCCAACGAGCTGCACTGCTATGACGTGGACTTCC<br>AGACCTGGGAGGTCGTCCAGCCCAGCTCCGACAGCGAGGTTGGTGGGGCTGAAGTGCCCGAGCGAGCCTGTGCTTCCGAGGAGGTGCCCACCCTGACCTATGAGG<br>AGCGGGTTGGCTTCAAGAAGTCCCGAGATGTGTTTGGCCTGGACTTTGGCACCACCTCAGCCAAGCAGCCCACCCAGCCTGCCTCGGAGCTGCCCAGTGGGAGGCT<br>CTTCCACGCGGCTGCTGTCATCTCGGACGCCATGTACATCTTCGGGGGCACGGTGGACAACAACATCCGCAGCGGGGAGATGTACAGGTTCTTCTCCTGTTACCCTA<br>AATGCACGCTGCACGAGGACTACGGGCGGCTGTGGGAGAGCCGCCAGTTCTGCGACGTGGAGTTCGTGCTGGGTGAGAAGGAGGAGTGCGTGCAGGGCCACGTAG<br>CCATTGTCACAGCGCGGAGCCGCTGGCTTCGCAGGAAGATCACGCAGGCGCGGGAGAGGCTGGCCCAGAAGCTGGAGCAGGAGGCCGCCCCAGTTCCCAGGGAGG<br>CCCCCGGCGTGGCTGCTGGTGGGGCCCGGCCGCCCCTGCTGCACGTGGCCATCCGGGAGGCCGAGGCCCGGCCCTTCGAGGTGCTCATGCAGTTCCTCTACACCGA<br>CAAGATCAAATACCCACGGAAAGGCCATGTGGAGGATGTGCTGCTCATCATGGATGTGTACAAACTGGCACTGAGCTTCCAGTTGTGCCGCCTGGAGCAGCTGTGC<br>CGCCAGTACATCGAGGCCTCCGTGGACCTGCAGAACGTGCTGGTTGTGTGCGAGAGTGCCGCCCGGCTGCAGCTGAGCCAACTCAAGGAGCACTGCCTGAACTTCG<br>TGGTAAAGGAGTCCCACTTCAACCAGGTGATCATGATGAAGGAGTTCGAGCGCCTCTCCTCTCCACTGATAGTGGAGATTGTGCGGCGGAAGCAGCAGCCGCCCCC<br>TCGCACTCCCTTGGACCAGCCAGTGGACATTGGCACATCTCTGATCCAGGACATGAAGGCATACCTGGAGGGAGCGGGCGCGGAATTCTGTGACATCACTCTGTTG<br>CTTGACGGGCACCCACGGCCAGCCCACAAGGCTATCCTGGCCGCCCGCTCCAGCTACTTTGAAGCCATGTTCCGGTCCTTCATGCCCGAAGATGGGCAGGTGAACA<br>TCTCCATCGGGGAGATGGTGCCCAGCAGGCAGGCCTTCGAGTCCATGCTGCGCTACATCTACTACGGCGAGGTCAACATGCCGCCCGAGGACTCGCTCTACTTGTTT<br>GCGGCCCCCTACTACTACGGCTTCTACAACAACCGGCTGCAGGCGTACTGCAAGCAGAACCTGGAGATGAACGTGACGGTGCAGAACGTGCTGCAGATCCTGGAG<br>GCAGCTGACAAAACGCAGGCACTGGACATGAAGCGGCACTGCCTGCACATCATTGTGCACCAGTTCACCAAGGTCTCCAAGTTGCCCACCCTGCGGTCGCTGAGCC<br>AGCAGCTGCTGCTGGACATCATAGACTCCCTGGCCTCCCACATCTCAGACAAGCAGTGCGCAGAGCTGGGCGCCGACATCTGA
**Aminoacid**	MAGPGSTGGQIGAAALAGGARSKVAPSVDFDHSCSDSVEYLTLNFGPFETVHRWRRLPPCDEFVGARRSKHTVVAYKDAIYVFGGDNGKTMLNDLLRFDVKDCSWCRA<br>FTTGTPPAPRYHHSAVVYGSSMFVFGGYTGDIYSNSNLKNKNDLFEYKFATGQWTEWKIEGRLPVARSAHGATVYSDKLWIFAGYDGNARLNDMWTIGLQDRELTCWE<br>EVAQSGEIPPSCCNFPVAVCRDKMFVFSGQSGAKITNNLFQFEFKDKTWTRIPTEHLLRGSPPPPQRRYGHTMVAFDRHLYVFGGAADNTLPNELHCYDVDFQTWEVVQP<br>SSDSEVGGAEVPERACASEEVPTLTYEERVGFKKSRDVFGLDFGTTSAKQPTQPASELPSGRLFHAAAVISDAMYIFGGTVDNNIRSGEMYRFFSCYPKCTLHEDYGRLWE<br>SRQFCDVEFVLGEKEECVQGHVAIVTARSRWLRRKITQARERLAQKLEQEAAPVPREAPGVAAGGARPPLLHVAIREAEARPFEVLMQFLYTDKIKYPRKGHVEDVLLIM<br>DVYKLALSFQLCRLEQLCRQYIEASVDLQNVLVVCESAARLQLSQLKEHCLNFVVKESHFNQVIMMKEFERLSSPLIVEIVRRKQQPPPRTPLDQPVDIGTSLIQDMKAYLE<br>GAGAEFCDITLLLDGHPRPAHKAILAARSSYFEAMFRSFMPEDGQVNISIGEMVPSRQAFESMLRYIYYGEVNMPPEDSLYLFAAPYYYGFYNNRLQAYCKQNLEMNVTV<br>QNVLQILEAADKTQALDMKRHCLHIIVHQFTKVSKLPTLRSLSQQLLLDIIDSLASHISDKQCAELGADI

	MT3 Sequences
**Donor splice site in exon 11th or its region**	GGTTCCATTTCT
**CDS**	ATGGCTGGACCGGGCAGCACGGGGGGGCAGATCGGGGCTGCGGCCCTGGCAGGCGGCGCGCGGTCCAAGGTAGCCCCGAGCGTGGACTTCGACCATAGCTGCTCG<br>GACAGTGTCGAGTACCTGACGCTCAACTTCGGGCCCTTCGAAACAGTGCATCGCTGGCGGCGCCTCCCGCCCTGCGACGAGTTCGTGGGTGCCCGGCGCAGCAAGC<br>ACACAGTGGTGGCCTATAAAGATGCCATTTATGTATTTGGTGGAGACAATGGGAAGACCATGCTCAATGACCTCCTGCGGTTCGATGTGAAAGACTGCTCCTGGTG<br>CAGGGCCTTTACCACTGGGACCCCACCGGCCCCCCGTTACCACCACTCGGCCGTCGTCTATGGGAGCAGCATGTTTGTCTTTGGGGGTTACACTGGGGACATTTATT<br>CCAATTCTAACTTGAAGAATAAAAACGACCTCTTTGAATACAAGTTTGCAACTGGCCAGTGGACGGAGTGGAAAATTGAAGGACGGTTGCCAGTCGCTAGGTCAGC<br>CCATGGGGCCACGGTGTACAGTGACAAGCTGTGGATCTTTGCTGGCTATGACGGCAACGCCAGGTTGAATGACATGTGGACAATTGGCCTCCAGGACCGAGAGCTC<br>ACCTGCTGGGAGGAGGTGGCCCAGAGTGGCGAGATCCCCCCATCTTGCTGCAACTTCCCCGTGGCTGTGTGCCGGGACAAGATGTTTGTATTCTCTGGGCAAAGCG<br>GAGCCAAAATAACCAACAACCTCTTCCAGTTTGAATTCAAGGACAAGACGTGGACACGCATCCCAACTGAACACCTGCTCCGGGGCTCCCCACCACCCCCGCAGCG<br>GCGCTACGGGCATACCATGGTGGCCTTTGACCGCCACCTCTATGTGTTTGGGGGTGCGGCCGACAACACGCTGCCCAACGAGCTGCACTGCTATGACGTGGACTTCC<br>AGACCTGGGAGGTCGTCCAGCCCAGCTCCGACAGCGAGGTTGGTGGGGCTGAAGTGCCCGAGCGAGCCTGTGCTTCCGAGGAGGTGCCCACCCTGACCTATGAGG<br>AGCGGGTTGGCTTCAAGAAGTCCCGAGATGTGTTTGGCCTGGACTTTGGCACCACCTCAGCCAAGCAGCCCACCCAGCCTGCCTCGGAGCTGCCCAGTGGGAGGCT<br>CTTCCACGCGGCTGCTGTCATCTCGGACGCCATGTACATCTTCGGGGGCACGGTGGACAACAACATCCGCAGCGGGGAGATGTACAGGTTCTTCTCCTGTTACCCTA<br>AATGCACGCTGCACGAGGACTACGGGCGGCTGTGGGAGAGCCGCCAGTTCTGCGACGTGGAGTTCGTGCTGGGTGAGAAGGAGGAGTGCGTGCAGGGCCACGTAG<br>CCATTGTCACAGCGCGGAGCCGCTGGCTTCGCAGGAAGATCACGCAGGCGCGGGAGAGGCTGGCCCAGAAGCTGGAGCAGGAGGCCGCCCCAGTTCCCAGGGAGG<br>CCCCCGGCGTGGCTGCTGGTGGGGCCCGGCCGCCCCTGCTGCACGTGGCCATCCGGGAGGCCGAGGCCCGGCCCTTCGAGGTGCTCATGCAGTTCCTCTACACCGA<br>CAAGATCAAATACCCACGGAAAGGCCATGTGGAGGATGTGCTGCTCATCATGGATGTGTACAAACTGGCACTGAGCTTCCAGTTGTGCCGCCTGGAGCAGCTGTGC<br>CGCCAGTACATCGAGGCCTCCGTGGACCTGCAGAACGTGCTGGTTGTGTGCGAGAGTGCCGCCCGGCTGCAGCTGAGCCAACTCAAGGAGCACTGCCTGAACTTCG<br>TGGTAAAGGAGTCCCACTTCAACCAGGTGATCATGATGAAGGAGTTCGAGCGCCTCTCCTCTCCACTGATAGTGGAGATTGTGCGGCGGAAGCAGCAGCCGCCCCC<br>TCGCACTCCCTTGGACCAGCCAGTGGACATTGGCACATCTCTGATCCAGGACATGAAGGCATACCTGGAGGGAGCGGGCGCGGAATTCTGTGACATCACTCTGTTG<br>CTTGACGGGCACCCACGGCCAGCCCACAAGGCTATCCTGGCCGCCCGCTCCAGCTACTTTGAAGCCATGTTCCGGTCCTTCATGCCCGAAGATGGGCAGGTGAACA<br>TCTCCATCGGGGAGATGGTGCCCAGCAGGCAGGCCTTCGAGTCCATGCTGCGCTACATCTACTACGGCGAGGTCAACATGCCGCCCGAGGACTCGCTCTACTTGTTT<br>GCGGCCCCCTACTACTACGGCTTCTACAACAACCGGCTGCAGGCGTACTGCAAGCAGAACCTGGAGATGAACGTGACGGTGCAGAACGTGCTGCAGATCCTGGAG<br>GCAGCTGACAAAACGCAGGCACTGGACATGAAGCGGCACTGCCTGCACATCATTGTGCACCAGTTCACCAAGGTCTCCAAGTTGCCCACCCTGCGGTCGCTGAGCC<br>AGCAGCTGCTGCTGGACATCATAGACTCCCTGGCCTCCCACATCTCAGACAAGCAGTGCGCAGAGCTGGGCGCCGACATCTGA
**Aminoacid**	MAGPGSTGGQIGAAALAGGARSKVAPSVDFDHSCSDSVEYLTLNFGPFETVHRWRRLPPCDEFVGARRSKHTVVAYKDAIYVFGGDNGKTMLNDLLRFDVKDCSWCRA<br>FTTGTPPAPRYHHSAVVYGSSMFVFGGYTGDIYSNSNLKNKNDLFEYKFATGQWTEWKIEGRLPVARSAHGATVYSDKLWIFAGYDGNARLNDMWTIGLQDRELTCWE<br>EVAQSGEIPPSCCNFPVAVCRDKMFVFSGQSGAKITNNLFQFEFKDKTWTRIPTEHLLRGSPPPPQRRYGHTMVAFDRHLYVFGGAADNTLPNELHCYDVDFQTWEVVQP<br>SSDSEVGGAEVPERACASEEVPTLTYEERVGFKKSRDVFGLDFGTTSAKQPTQPASELPSGRLFHAAAVISDAMYIFGGTVDNNIRSGEMYRFHSPVTLNARCTRTTGGCG<br>RAASSATWSSCWVRRRSACRAT

	MT4 Sequences
**Donor splice site in exon 11th or its region**	TGTGGGGCCTGT
**CDS**	ATGGCTGGACCGGGCAGCACGGGGGGGCAGATCGGGGCTGCGGCCCTGGCAGGCGGCGCGCGGTCCAAGGTAGCCCCGAGCGTGGACTTCGACCATAGCTGCTCG<br>GACAGTGTCGAGTACCTGACGCTCAACTTCGGGCCCTTCGAAACAGTGCATCGCTGGCGGCGCCTCCCGCCCTGCGACGAGTTCGTGGGTGCCCGGCGCAGCAAGC<br>ACACAGTGGTGGCCTATAAAGATGCCATTTATGTATTTGGTGGAGACAATGGGAAGACCATGCTCAATGACCTCCTGCGGTTCGATGTGAAAGACTGCTCCTGGTG<br>CAGGGCCTTTACCACTGGGACCCCACCGGCCCCCCGTTACCACCACTCGGCCGTCGTCTATGGGAGCAGCATGTTTGTCTTTGGGGGTTACACTGGGGACATTTATT<br>CCAATTCTAACTTGAAGAATAAAAACGACCTCTTTGAATACAAGTTTGCAACTGGCCAGTGGACGGAGTGGAAAATTGAAGGACGGTTGCCAGTCGCTAGGTCAGC<br>CCATGGGGCCACGGTGTACAGTGACAAGCTGTGGATCTTTGCTGGCTATGACGGCAACGCCAGGTTGAATGACATGTGGACAATTGGCCTCCAGGACCGAGAGCTC<br>ACCTGCTGGGAGGAGGTGGCCCAGAGTGGCGAGATCCCCCCATCTTGCTGCAACTTCCCCGTGGCTGTGTGCCGGGACAAGATGTTTGTATTCTCTGGGCAAAGCG<br>GAGCCAAAATAACCAACAACCTCTTCCAGTTTGAATTCAAGGACAAGACGTGGACACGCATCCCAACTGAACACCTGCTCCGGGGCTCCCCACCACCCCCGCAGCG<br>GCGCTACGGGCATACCATGGTGGCCTTTGACCGCCACCTCTATGTGTTTGGGGGTGCGGCCGACAACACGCTGCCCAACGAGCTGCACTGCTATGACGTGGACTTCC<br>AGACCTGGGAGGTCGTCCAGCCCAGCTCCGACAGCGAGGTTGGTGGGGCTGAAGTGCCCGAGCGAGCCTGTGCTTCCGAGGAGGTGCCCACCCTGACCTATGAGG<br>AGCGGGTTGGCTTCAAGAAGTCCCGAGATGTGTTTGGCCTGGACTTTGGCACCACCTCAGCCAAGCAGCCCACCCAGCCTGCCTCGGAGCTGCCCAGTGGAGGCT<br>CTTCCACGCGGCTGCTGTCATCTCGGACGCCATGTACATCTTCGGGGGCACGGTGGACAACAACATCCGCAGCGGGGAGATGTACAGGTTCCAGTGTGGGGCCGCA<br>GCGGCGCTACGGGCATACCATGGTGGCCTTTGACCGCCACCTCTATGTGTTTGGGGGTGCGGCCGACAACACGCTGCCCAACGAGCTGCACTGCTATGACGTGGAC<br>TTCCAGACCTGGGAGGTCGTCCAGCCCAGCTCCGACAGCGAGGTTGGTGGGGCTGAAGTGCCCGAGCGAGCCTGTGCTTCCGAGGAGGTGCCCACCCTGACCTATG<br>AGGAGCGGGTTGGCTTCAAGAAGTCCCGAGATGTGTTTGGCCTGGACTTTGGCACCACCTCAGCCAAGCAGCCCACCCAGCCTGCCTCGGAGCTGCCCAGTGGGAG<br>GCTCTTCCACGCGGCTGCTGTCATCTCGGACGCCATGTACATCTTCGGGGGCACGGTGGACAACAACATCCGCAGCGGGGAGATGTACAGGTTCCAGTGTGGGGTT<br>CTCCTGTTACCCTAAATGCACGCTGCACGAGGACTACGGGCGGCTGTGGGAGAGCCGCCAGTTCTGCGACGTGGAGTTCGTGCTGGGTGAGAAGGAGGAGTGCGTG<br>CAGGGCCACGTAGCCATTGTCACAGCGCGGAGCCGCTGGCTTCGCAGGAAGATCACGCAGGCGCGGGAGAGGCTGGCCCAGAAGCTGGAGCAGGAGGCCGCCCC<br>AGTTCCCAGGGAGGCCCCCGGCGTGGCTGCTGGTGGGGCCCGGCCGCCCCTGCTGCACGTGGCCATCCGGGAGGCCGAGGCCCGGCCCTTCGAGGTGCTCATGCAG<br>TTCCTCTACACCGACAAGATCAAATACCCACGGAAAGGCCATGTGGAGGATGTGCTGCTCATCATGGATGTGTACAAACTGGCACTGAGCTTCCAGTTGTGCCGCC<br>TGGAGCAGCTGTGCCGCCAGTACATCGAGGCCTCCGTGGACCTGCAGAACGTGCTGGTTGTGTGCGAGAGTGCCGCCCGGCTGCAGCTGAGCCAACTCAAGGAGCA<br>CTGCCTGAACTTCGTGGTAAAGGAGTCCCACTTCAACCAGGTGATCATGATGAAGGAGTTCGAGCGCCTCTCCTCTCCACTGATAGTGGAGATTGTGCGGCGGAAG<br>CAGCAGCCGCCCCCTCGCACTCCCTTGGACCAGCCAGTGGACATTGGCACATCTCTGATCCAGGACATGAAGGCATACCTGGAGGGAGCGGGCGCGGAATTCTGTG<br>ACATCACTCTGTTGCTTGACGGGCACCCACGGCCAGCCCACAAGGCTATCCTGGCCGCCCGCTCCAGCTACTTTGAAGCCATGTTCCGGTCCTTCATGCCCGAAGAT<br>GGGCAGGTGAACATCTCCATCGGGGAGATGGTGCCCAGCAGGCAGGCCTTCGAGTCCATGCTGCGCTACATCTACTACGGCGAGGTCAACATGCCGCCCGAGGACT<br>CGCTCTACTTGTTTGCGGCCCCCTACTACTACGGCTTCTACAACAACCGGCTGCAGGCGTACTGCAAGCAGAACCTGGAGATGAACGTGACGGTGCAGAACGTGCT<br>GCAGATCCTGGAGGCAGCTGACAAAACGCAGGCACTGGACATGAAGCGGCACTGCCTGCACATCATTGTGCACCAGTTCACCAAGGTCTCCAAGTTGCCCACCCTG
**Aminoacid**	MRSGLASRSPEMCLAWTLAPPQPSSPPSLPRSCPVGGSSTRLLSSRTPCTSSGARWTTTSAAGRCTGSSVGFSCYPKCTLHEDYGRLWESRQFCDVEFVLGEKEECVQGHV<br>AIVTARSRWLRRKITQARERLAQKLEQEAAPVPREAPGVAAGGARPPLLHVAIREAEARPFEVLMQFLYTDKIKYPRKGHVEDVLLIMDVYKLALSFQLCRLEQLCRQYIE<br>ASVDLQNVLVVCESAARLQLSQLKEHCLNFVVKESHFNQVIMMKEFERLSSPLIVEIVRRKQQPPPRTPLDQPVDIGTSLIQDMKAYLEGAGAEFCDITLLLDGHPRPAHKA<br>ILAARSSYFEAMFRSFMPEDGQVNISIGEMVPSRQAFESMLRYIYYGEVNMPPEDSLYLFAAPYYYGFYNNRLQAYCKQNLEMNVTVQNVLQILEAADKTQALDMKRHC<br>LHIIVHQFTKVSKLPTLRSLSQQLLLDIIDSLASHISDKQCAELGADI
