# Supplementary material for: LZTR1: c.1260+1del Variant as a Significant Predictor of Early-Age Breast Cancer Development: Case Report Combined with In Silico Analysis
Source: Int J Mol Sci. 2025 Jul 12;26(14):6704. doi: 10.3390/ijms26146704 (PMC12294705; doi:10.3390/ijms26146704)
Supplement: Supplementary file 1 [file ijms-26-06704-s001.zip › supplementary S3 (LZTR1 case study).pdf]

Supmat (BDGP) Splice site prediction MT and WT LZTR1

Donor site predictions for LZTR1WT :				Acceptor site predictions for LZTR1WT:			
Start	End	Score	Exon Intron	Start	End	Score	Intron Exon
138	152	0.79	gtccaaggtagcccc	562	602	0.41	gtcaggggtcctcacttgcaggatgattggtgttatcttag
269	283	0.80	gtgcccggtacggtg	669	709	0.53	cattccttgggtgccccccaggagggtcctgtccttaccgc
521	535	0.61	ctctgaggtggggtg	710	750	0.99	cctccactcctttctttccaggcgcagcaagcacacagtgg
787	801	0.99	acaatgggtgagtga	866	906	0.92	gctccattactgtcctttcagatgctagctggtgcctctaa
820	834	0.94	ggaccaggtagggag	1033	1073	0.72	aaatgtccctcttctgagtagatgcagccttgaaagaaaca
951	965	0.75	ggtcgtggtgaggat	1384	1424	0.54	ggcctcttactggctctgcagccttggtcctgtaggcctcc
1122	1136	0.80	ctcatcggtatggag	1398	1438	0.65	tctgcagccttggtcctgtaggcctccttgggggggcgggct
2151	2165	0.64	ttagcaggtagtttg	1532	1572	0.99	cctcccactctctttccccaggagtagccggtggggcccat
2304	2318	0.73	ggggcaagtaaggag	1667	1707	0.85	ctgctgctcctctgccctcagtccctcactgcactggggcg
2427	2441	0.90	aatggaggtgagaga	1916	1956	0.70	ggttattatccatctcttcagtcaaggcagggcctgtgctc
3595	3609	0.72	ggtgcaggtgggtgg	2137	2177	0.79	cctcaagagtgtccttagcaggtagtttgtgtgacaactgt
3778	3792	0.45	agatcaagtaaccac	2336	2376	0.43	acaatcaccaccagcttctaggctcctggcttcctggctgc
4040	4054	0.84	caacaaagtgagacc	2523	2563	0.83	cagtttgcccttcttcccaagggctgggtagtgatatgcca
4170	4184	0.51	atgataggtagatta	2765	2805	0.98	ttttttttttttttttttgagatggagtcttgctctgtcac
4246	4260	0.62	atgataggtagatga	2925	2965	0.97	ctaatttttttgtatttttagtagagatggggtttcaccat
4257	4271	0.98	atgataggtaggtag	2996	3036	0.43	tcaagtgatccgcccgcctaggcttcccaaagtgctgggat
4317	4331	0.65	atgataggtagatag	3069	3109	0.98	tctctatttcttttttttaagaagtggcaggaagatcagag
4363	4377	0.52	tatataggtagatga	3137	3177	0.48	gggcaagcacttgcattgcagggtacagctgcagaggaagg
4429	4443	0.65	atgataggtagatag	3247	3287	0.64	catttcatgccttcatgtcagctgctggttgggctcctggt
4564	4578	0.74	acattctgtgagtgg	3298	3338	0.87	atgctttggtcctgaccccaggcaggctggtgtctgcctgt
4650	4664	0.56	aggagaggtgtgcag	3524	3564	0.94	tctcttccggctgcctttcaggaagaccatgctcaatgacc
4691	4705	0.42	ttttctggtaatggg	3622	3662	0.94	gggccctgcctttcctcctagaacacagtggcacagtgctg
4778	4792	0.86	gagacaggtatcacc	4747	4787	0.97	tggtgattttttttttttaagtttctatgatgagacaggta
4878	4892	0.84	acctgaggtcagaac	5187	5227	0.79	ccacccctgtgcccaccccagggcctttaccactgggaccc
4958	4972	0.53	catgatggtgggtgc	5290	5330	0.95	aagcagcctcttgcctcccaggggctgtgtcgccccgaggc
5281	5295	0.99	gtctttggtaagcag	5335	5375	0.97	agacaccctgcccttctgcaggcctggggcatttgtgctcg
5816	5830	0.95	aaggacggtgagaaa	5531	5571	0.80	tgctcatctctgtgtccccagagcccagcacagaggcagac
6071	6085	0.61	agccgaggtgggaag	5606	5646	0.71	tcctggcttatgcattttcaggggggccagattctgctcca
6501	6515	0.78	tcgctaggtcagccc	5634	5674	0.90	agattctgctccaccttccagggtttgaaatctccaagact
6570	6584	0.51	acgccaggtgggtgg	5693	5733	0.94	tttctcactctctttactcaggggggttacactggggacatt
6750	6764	0.90	gatggaggtgagggc	5885	5925	0.98	ctgcccccttttgcctcccagatgaggaccctgagggcacg
7380	7394	0.69	ggaggaggtgagggg	5977	6017	0.64	tggcctgctcccagcccttagactccctcctttcagaagag
7408	7422	0.41	ggcgcaggtagagga	5992	6032	0.91	ccttagactccctcctttcagaagagacccaaatggaccag
7417	7431	0.87	agaggaggtgagggg	6327	6367	0.69	ggggctgttgtttattcctagtgccttgtggaggtcctgaa
7454	7468	0.69	ggaggaggtgagggg	6472	6512	0.95	ccctcttccctcacactccaggttgccagtcgctaggtcag
8223	8237	0.99	acaagacgtgagtac	6487	6527	0.52	ctccaggttgccagtcgctaggtcagcccatggggccacgg
8258	8272	0.76	gaggacggtcagttc	6875	6915	0.62	ccagccctggttcttgcccaggccaaaggccttttcctcct
8316	8330	0.48	ggccatggtaaccat	6903	6943	0.90	gccttttcctccttattttagaaagttggctttgctctctt
8497	8511	0.73	tagcttggtcagtgc	7036	7076	0.96	ctgtgtcctctccccacccaggagggacactgagtgaggag
8667	8681	0.83	gctgcaggtacaggg	7308	7348	0.86	cactggtctgtcctaatacaggttgaatgacatgtggacaa
8715	8729	0.49	gccacgggtgcgtgg	7698	7738	0.64	ttaacgcaccctttatttcagtgcagtgagggccgtctgct
8865	8879	0.65	ctgggaggtactggg	7769	7809	0.86	tgggcttgctttcccctttagaaacatggaatttatttatt
9220	9234	0.93	agtcaccgtaaggga	8069	8109	0.99	agctgtcctctccccctgcaggtggcccagagtggcgagat
9275	9289	0.95	cccgagggtgagcag	8479	8519	0.44	cgtccccttcctcgtgaatagcttggtcagtgccaccagta
9527	9541	0.97	cagcgaggtgagggt	9006	9046	0.77	ctgcacaactttactccccagagagcaggactgcctgggcg
9721	9735	0.76	cctgcaggtagttaa	9311	9351	0.83	ttcactgttgtgtacccccaggtggacacgcatcccaactg
10067	10081	0.79	ctcggaggtacaggc	9592	9632	0.96	ctcccacccagctcctcacagctttggggccccctggggtt
10602	10616	0.79	gttccaggtgtgggg	9700	9740	0.65	agccccagcttcaccccacagcctgcaggtagttaacgctt
10993	11007	0.61	cagtgaggtcagggc	9805	9845	0.96	tgcagctcttccttctttcagaacccactctcaaggccagg
11043	11057	0.45	ggccagtgtgaggac	9897	9937	0.99	ccatgtgtctcccctcttcaggttggtggggctgaagtgcc
11452	11466	0.60	gggtgaggtgggtgc	10099	10139	0.62	ctccatcaccccctgaaacaggtcctgtgatcaacacctct
11717	11731	0.95	ggcccaggtgaggtg	10338	10378	0.72	cccacctgtgtctgtacccaggggccctcatcccttgggga
12031	12045	0.96	ccaaaaggtgggtgc	10477	10517	0.98	ccctgccttcttgtcccccagctgcccagtgggaggctctt
12425	12439	0.83	actcaaggtgtgggg	10932	10972	0.73	gcgttgacagccccttcccaggcctggaggagcaggtggtc
12499	12513	0.60	caccactgtgagccc	11218	11258	0.86	ctgcttcctctcctcagccaggcccacctgcctcctgggcc
12588	12602	0.57	cttcgtggtaaagga	11345	11385	0.92	ctcactgggcccctcttgcagttctcctgttaccctaaatg
12612	12626	0.69	caaccaggtgatcat	11607	11647	0.78	agtgcatcattctttgtgcagaaggaggagtgcgtgcaggg
13182	13196	0.68	tcatgaggtcagcga	11787	11827	0.96	tgagctcccttctccccacagaagctggagcaggaggccgc
13376	13390	0.59	caggaaggtagtgcc	12144	12184	0.56	cgaggccttgttcctacctagtggccccagcccacactctt
13570	13584	0.69	gctccaggtgggtgg	12553	12593	0.51	gcctgtgcctgtctgccccaggagcactgcctgaacttcgt
13636	13650	0.45	cggccaggtgcctac	12981	13021	0.50	ggggcacctctttcgggctaggatgtcaggtctgatggccc
14099	14113	0.73	catggaggtactcct	13051	13091	0.47	tctttgcttgtaatgtcacaggccccttcattcccccagct
14499	14513	0.61	gctgcaggtagcccc	13069	13109	0.94	caggccccttcattcccccagctctccctggggcttgtgct
14664	14678	0.88	caccaaggtcagggc	13123	13163	0.71	ggctcttggtgatgtctgcaggcaccagaggccatgcagtg
14847	14861	0.72	gagcagggtaggccc	13411	13451	0.42	tcccaggctgtacctgctcagggaccctcctacccccaggc
15537	15551	0.95	gatccatgtaagggg	13429	13469	0.67	cagggaccctcctacccccaggcacatctctgatccaggac
15567	15581	0.77	ggaacttgtgagtgg	13556	13596	0.80	tatcctggccgcccgctccaggtgggtgggggctggacagg
15609	15623	0.63	acttgaggtgaattt	13646	13686	0.87	cctaccgctcgttgtctgcagctactttgaagccatgttcc
15636	15650	0.75	ccctgaggtcagctc	13813	13853	0.59	cgctgcatcctcactccccagtgaactcccaggtccccacc
15898	15912	0.75	gatgtaggtcagtgg	13857	13897	0.73	gggtcctggcacccacctcaggtggctttgaggcccgctct
16256	16270	0.81	tatgcaggtgtgtgg	13891	13931	0.77	ccgctctcctgccccagctaggtgatctgggcccagcgcct

16408	16422	0.99	ccttgaggtgaggac	14067	14107	0.62	ggtcattgctttgtgtgacagttgagcacagacatggaggt
				14109	14149	0.80	ctccttatgtcacttcagtaggggtctgaacatggagggtc
				14675	14715	0.90	gggctctggcctccccttcaggactcgcttccccttggcag
				14695	14735	0.80	ggactcgcttccccttggcagtggccatgcccaggcaggga
				14915	14955	0.90	tccggcctgcttgccttacaggtctccaagttgcccaccct
				14997	15037	0.73	ccctggcctcccacatctcagacaagcagtgcgcagagctg
				15284	15324	0.71	catcaccctctcctggtgtagtgtggatgcgaggccacggc
				15668	15708	0.55	ggccaggcctggttttcacaggggctgaaggatcccagtcc
				15705	15745	0.87	gtccacctgtgtgcatgtcagggctcggccgggaagaagcc
				15993	16033	0.58	ctccaggctggccccttccaggggaatcctggaggcctggg
				16097	16137	0.51	agggctgccctcttgccctagtgagggccccatgtggatcc
				16242	16282	0.82	ctctcctttgggtgtatgcaggtgtgtgggggggccctgagt

Donor site predictions for LZTR1MT :

Start	End	Score	Exon Intron
138	152	0.79	gtccaaggtagcccc
269	283	0.80	gtgcccggtacggtg
521	535	0.61	ctctgaggtggggtg
787	801	0.99	acaatgggtgagtga
820	834	0.94	ggaccaggtagggag
951	965	0.75	ggtcgtggtgaggat
1122	1136	0.80	ctcatcggtatggag
2151	2165	0.64	ttagcaggtagtttg
2304	2318	0.73	ggggcaagtaaggag
2427	2441	0.90	aatggaggtgagaga
3595	3609	0.72	ggtgcaggtgggtgg
3778	3792	0.45	agatcaagtaaccac
4040	4054	0.84	caacaaagtgagacc
4170	4184	0.51	atgataggtagatta
4246	4260	0.62	atgataggtagatga
4257	4271	0.98	atgataggtaggtag
4317	4331	0.65	atgataggtagatag
4363	4377	0.52	tatataggtagatga
4429	4443	0.65	atgataggtagatag
4564	4578	0.74	acattctgtgagtgg
4650	4664	0.56	aggagaggtgtgcag
4691	4705	0.42	ttttctggtaatggg
4778	4792	0.86	gagacaggtatcacc
4878	4892	0.84	acctgaggtcagaac
4958	4972	0.53	catgatggtgggtgc
5281	5295	0.99	gtctttggtaagcag
5816	5830	0.95	aaggacggtgagaaa
6071	6085	0.61	agccgaggtgggaag
6501	6515	0.78	tcgctaggtcagccc
6570	6584	0.51	acgccaggtgggtgg
6750	6764	0.90	gatggaggtgagggc
7380	7394	0.69	ggaggaggtgagggg
7408	7422	0.41	ggcgcaggtagagga
7417	7431	0.87	agaggaggtgagggg
7454	7468	0.69	ggaggaggtgagggg
8223	8237	0.99	acaagacgtgagtac
8258	8272	0.76	gaggacggtcagttc
8316	8330	0.48	ggccatggtaaccat
8497	8511	0.73	tagcttggtcagtgc
8667	8681	0.83	gctgcaggtacaggg
8715	8729	0.49	gccacgggtgcgtgg
8865	8879	0.65	ctgggaggtactggg
9220	9234	0.93	agtcaccgtaaggga
9275	9289	0.95	cccgagggtgagcag
9527	9541	0.97	cagcgaggtgagggt
9721	9735	0.76	cctgcaggtagttaa
10067	10081	0.79	ctcggaggtacaggc
10994	11008	0.61	cagtgaggtcagggc
11044	11058	0.45	ggccagtgtgaggac
11453	11467	0.60	gggtgaggtgggtgc
11718	11732	0.95	ggcccaggtgaggtg
12032	12046	0.96	ccaaaaggtgggtgc
12426	12440	0.83	actcaaggtgtgggg
12500	12514	0.60	caccactgtgagccc
12589	12603	0.57	cttcgtggtaaagga
12613	12627	0.69	caaccaggtgatcat
13183	13197	0.68	tcatgaggtcagcga
13377	13391	0.59	caggaaggtagtgcc
13571	13585	0.69	gctccaggtgggtgg
13637	13651	0.45	cggccaggtgcctac

Acceptor site predictions for LZTR1MT:

Start	End	Score	Intron Exon
562	602	0.41	gtcaggggtcctcacttgcaggatgattggtgttatcttag
669	709	0.53	cattccttgggtgccccccaggagggtcctgtccttaccgc
710	750	0.99	cctccactcctttctttccaggcgcagcaagcacacagtgg
866	906	0.92	gctccattactgtcctttcagatgctagctggtgcctctaa
1033	1073	0.72	aaatgtccctcttctgagtagatgcagccttgaaagaaaca
1384	1424	0.54	ggcctcttactggctctgcagccttggtcctgtaggcctcc
1398	1438	0.65	tctgcagccttggtcctgtaggcctccttgggggggcgggct
1532	1572	0.99	cctcccactctctttccccaggagtagccggtggggcccat
1667	1707	0.85	ctgctgctcctctgccctcagtccctcactgcactggggcg
1916	1956	0.70	ggttattatccatctcttcagtcaaggcagggcctgtgctc
2137	2177	0.79	cctcaagagtgtccttagcaggtagtttgtgtgacaactgt
2336	2376	0.43	acaatcaccaccagcttctaggctcctggcttcctggctgc
2523	2563	0.83	cagtttgcccttcttcccaagggctgggtagtgatatgcca
2765	2805	0.98	ttttttttttttttttttgagatggagtcttgctctgtcac
2925	2965	0.97	ctaatttttttgtatttttagtagagatggggtttcaccat
2996	3036	0.43	tcaagtgatccgcccgcctaggcttcccaaagtgctgggat
3069	3109	0.98	tctctatttcttttttttaagaagtggcaggaagatcagag
3137	3177	0.48	gggcaagcacttgcattgcagggtacagctgcagaggaagg
3247	3287	0.64	catttcatgccttcatgtcagctgctggttgggctcctggt
3298	3338	0.87	atgctttggtcctgaccccaggcaggctggtgtctgcctgt
3524	3564	0.94	tctcttccggctgcctttcaggaagaccatgctcaatgacc
3622	3662	0.94	gggccctgcctttcctcctagaacacagtggcacagtgctg
4747	4787	0.97	tggtgattttttttttttaagtttctatgatgagacaggta
5187	5227	0.79	ccacccctgtgcccaccccagggcctttaccactgggaccc
5290	5330	0.95	aagcagcctcttgcctcccaggggctgtgtcgccccgaggc
5335	5375	0.97	agacaccctgcccttctgcaggcctggggcatttgtgctcg
5531	5571	0.80	tgctcatctctgtgtccccagagcccagcacagaggcagac
5606	5646	0.71	tcctggcttatgcattttcaggggggccagattctgctcca
5634	5674	0.90	agattctgctccaccttccagggtttgaaatctccaagact
5693	5733	0.94	tttctcactctctttactcaggggggttacactggggacatt
5885	5925	0.98	ctgccccctttgcctcccagatgaggaccctgagggcacg
5977	6017	0.64	tggcctgctcccagcccttagactccctcctttcagaagag
5992	6032	0.91	ccttagactccctcctttcagaagagacccaaatggaccag
6327	6367	0.69	ggggctgttgtttattcctagtgccttgtggaggtcctgaa
6472	6512	0.95	ccctcttccctcacactccaggttgccagtcgctaggtcag
6487	6527	0.52	ctccaggttgccagtcgctaggtcagcccatggggccacgg
6875	6915	0.62	ccagccctggttcttgcccaggccaaaggccttttcctcct
6903	6943	0.90	gccttttcctccttattttagaaagttggctttgctctctt
7036	7076	0.96	ctgtgtcctctccccacccaggagggacactgagtgaggag
7308	7348	0.86	cactggtctgtcctaatacaggttgaatgacatgtggacaa
7698	7738	0.64	ttaacgcaccctttatttcagtgcagtgagggccgtctgct
7769	7809	0.86	tgggcttgctttcccctttagaaacatggaatttatttatt
8069	8109	0.99	agctgtcctctccccctgcaggtggcccagagtggcgagat
8479	8519	0.44	cgtccccttcctcgtgaatagcttggtcagtgccaccagta
9006	9046	0.77	ctgcacaactttactccccagagagcaggactgcctgggcg
9311	9351	0.83	ttcactgttgtgtacccccaggtggacacgcatcccaactg
9592	9632	0.96	ctcccacccagctcctcacagctttggggccccctggggtt
9700	9740	0.65	agccccagcttcaccccacagcctgcaggtagttaacgctt
9805	9845	0.96	tgcagctcttccttctttcagaacccactctcaaggccagg
9897	9937	0.99	ccatgtgtctcccctcttcaggttggtggggctgaagtgcc
10099	10139	0.62	ctccatcacccccctgaaacaggtcctgtgatcaacacctct
10338	10378	0.72	cccacctgtgtctgtacccaggggccctcatcccttgggga
10477	10517	0.98	ccctgccttcttgtcccccagctgcccagtgggaggctctt
10933	10973	0.73	gcgttgacagccccttcccaggcctggaggagcaggtggtc
11219	11259	0.86	ctgcttcctctcctcagccaggcccacctgcctcctgggcc
11346	11386	0.92	ctcactgggcccctcttgcagttctcctgttaccctaaatg
11608	11648	0.78	agtgcatcattctttgtgcagaaggaggagtgcgtgcaggg
11788	11828	0.96	tgagctcccttctccccacagaagctggagcaggaggccgc
12145	12185	0.56	cgaggccttgttcctacctagtggccccagcccacactctt
12554	12594	0.51	gcctgtgcctgtctgccccaggagcactgcctgaacttcgt

14100 14114	0.73	catggaggtactcct	12982 13022	0.50	ggggcacctctttcgggctaggatgtcaggtctgatggccc
14500 14514	0.61	gctgcaggtagcccc	13052 13092	0.47	tctttgcttgtaatgtcacaggccccttcattcccccagct
14665 14679	0.88	caccaaggtcagggc	13070 13110	0.94	caggccccttcattcccccagctctccctggggcttgtgct
14848 14862	0.72	gagcagggtaggccc	13124 13164	0.71	ggctcttggtgatgtctgcaggcaccagaggccatgcagtg
15538 15552	0.95	gatccatgtaagggg	13412 13452	0.42	tcccaggctgtacctgctcagggaccctcctacccccaggc
15568 15582	0.77	ggaacttgtgagtgg	13430 13470	0.67	cagggaccctcctacccccaggcacatctctgatccaggac
15610 15624	0.63	acttgaggtgaattt	13557 13597	0.80	tatcctggccgcccgctccaggtgggtgggggctggacagg
15637 15651	0.75	ccctgaggtcagctc	13647 13687	0.87	cctaccgctcgttgtctgcagctactttgaagccatgttcc
15899 15913	0.75	gatgtaggtcagtgg	13814 13854	0.59	cgctgcatcctcactccccagtgaactcccaggtccccacc
16257 16271	0.81	tatgcaggtgtgtgg	13858 13898	0.73	gggtcctggcacccacctcaggtggctttgaggcccgctct
16409 16423	0.99	ccttgaggtgaggac	13892 13932	0.77	ccgctctcctgccccagctaggtgatctgggcccagcgcct
			14068 14108	0.62	ggtcattgctttgtgtgacagttgagcacagacatggaggt
			14110 14150	0.80	ctccttatgtcacttcagtaggggtctgaacatggagggtc
			14676 14716	0.90	gggctctggcctccccttcaggactcgcttccccttggcag
			14696 14736	0.80	ggactcgcttccccttggcagtggccatgcccaggcaggga
			14916 14956	0.90	tccggcctgcttgccttacaggtctccaagttgcccaccct
			14998 15038	0.73	ccctggcctcccacatctcagacaagcagtgcgcagagctg
			15285 15325	0.71	catcaccctctcctggtgtagtgtggatgcgaggccacggc
			15669 15709	0.55	ggccaggcctggttttcacaggggctgaaggatcccagtcc
			15706 15746	0.87	gtccacctgtgtgcatgtcagggctcggccgggaagaagcc
			15994 16034	0.58	ctccaggctggccccttccaggggaatcctggaggcctggg
			16098 16138	0.51	agggctgccctcttgccctagtgagggccccatgtggatcc
			16243 16283	0.82	ctctcctttgggtgtatgcaggtgtgtggggggccctgagt
